# Ultrastable, supertough and photohealable polymer

**DOI:** 10.1093/nsr/nwaf521

**Published:** 2025-11-27

**Authors:** Zekai Wu, Yuhui Jin, Yingqian Li, Guangchen Liu, Zhengwei You

**Affiliations:** State Key Laboratory of Advanced Fibers Materials, Institute of Functional Materials, College of Materials Science and Engineering, Research Base of Textile Materials for Flexible Electronics and Biomedical Applications (China Textile Engineering Society), Shanghai Key Laboratory of Lightweight Composite, Shanghai Engineering Research Center of Nano-Biomaterials and Regenerative Medicine, Donghua University, Shanghai 201620, China; State Key Laboratory of Advanced Fibers Materials, Institute of Functional Materials, College of Materials Science and Engineering, Research Base of Textile Materials for Flexible Electronics and Biomedical Applications (China Textile Engineering Society), Shanghai Key Laboratory of Lightweight Composite, Shanghai Engineering Research Center of Nano-Biomaterials and Regenerative Medicine, Donghua University, Shanghai 201620, China; Shandong Inov Polyurethane Co., Ltd, Zibo 255000, China; Shandong Inov Polyurethane Co., Ltd, Zibo 255000, China; State Key Laboratory of Advanced Fibers Materials, Institute of Functional Materials, College of Materials Science and Engineering, Research Base of Textile Materials for Flexible Electronics and Biomedical Applications (China Textile Engineering Society), Shanghai Key Laboratory of Lightweight Composite, Shanghai Engineering Research Center of Nano-Biomaterials and Regenerative Medicine, Donghua University, Shanghai 201620, China

**Keywords:** metal-induced enhancement, polarized hydrogen bond, π–π interaction, photohealable polymer, elastomer

## Abstract

There is usually a mutual trade-off between thermodynamic stability, kinetic activity and external field responsiveness in polymer chemistry. Here, we report a Cu(II)-coordinated benzoquinone dioxime–carbamate unit (Cu–BQDU) that resolves this challenge in polymers. Through an inductive effect, coordination bonds polarize hydrogen bonds, enhancing their strength to the highest reported value for carbamate–carbamate segments in hydrogen bonding (∼5.6 kcal/mol). The resulting polymer exhibits exceptional thermodynamic stability and achieves a record-high toughness of 236.0 MJ/m^3^ among intrinsic photothermal elastomers. Additionally, the coordination bond extends the molecular conjugation length, optimizing the geometric alignment of π–π interactions to enhance photothermal conversion efficiency. This effect synergizes with Cu(II)-catalysed carbamate dynamics, boosting near-infrared-light-driven photothermal healing efficiency by 92.9%. This work provides multiple new molecular design principles of polymers.

## Teaser

Leveraging coordination bonds to simultaneously polarize H bonds, optimize π–π interactions and catalyse oxime–carbamate bonds achieves the simultaneous enhancement of elastomers’ mechanical properties, thermal stability, photothermal conversion efficiency and self-healing efficiency.

## INTRODUCTION

In the field of polymer materials, achieving the synergistic optimization of thermodynamic stability, kinetic activity and external field regulation has long been a significant scientific challenge [1–3]. The core of this contradiction lies in the fact that thermodynamic stability relies on low-free-energy equilibrium states formed by strong intermolecular interactions, whereas kinetic activity demands the sufficient mobility of molecular segments or networks to rapidly respond to external stimuli and maintain new metastable or equilibrium states post-regulation [4,5]. For instance, traditional cross-linked elastomers exhibit excellent thermodynamic stability, but suffer from rigid network structures that severely restrict segmental motion, leading to a sluggish dynamic response and insufficient external field regulation capability [6]. Conversely, dynamic covalent polymers can achieve responsiveness via reversible bond breakage and recombination, but often lack sufficient thermodynamic stability due to weak interactions or low bond energy, failing to meet the demands in complex environmental applications [7]. This inherent contradiction makes the simultaneous optimization of these three properties in a single material a formidable scientific challenge, particularly in materials requiring precise spatiotemporal control, for which breakthroughs in related technical bottlenecks are urgently needed [8,9].

Photohealable elastomers, which utilize light energy to drive self-healing after damage, offer a promising platform for polymer design that coordinates thermodynamic, kinetic and external field regulation performances [10,11]. However, in filler-dependent photo-healing systems, nonuniform filler distribution often introduces interfacial defects, disrupting the uniformity of tensile strength and photothermal conversion [12–14]. Intrinsic photo-healing systems avoid issues such as filler agglomeration and phase separation by incorporating photothermal-responsive groups into the polymer [10,15–18]. Nevertheless, this system faces the challenge of balancing the competing demands of polymer chain mobility for healing and structural stability under prolonged light exposure [14,17,19–22]. Overall, while highly desirable, integrating a material with three critical attributes—robust mechanical properties, high photothermal conversion efficiency and stable yet reconfigurable dynamic networks—remains a challenge.

**Figure 1.**
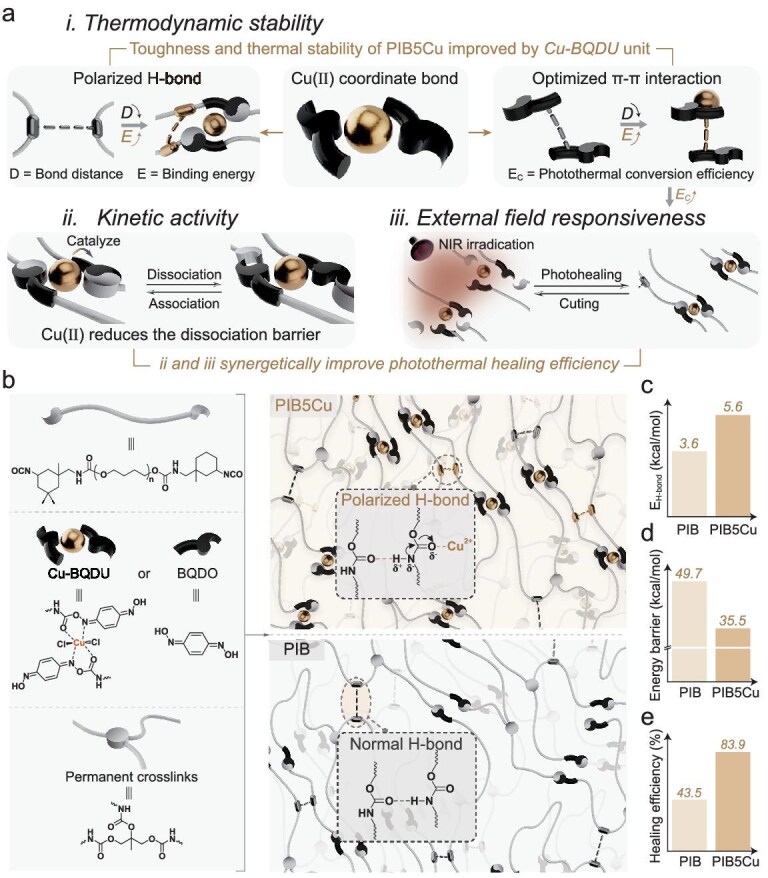
Design of PIB and PIB5Cu. (a) Schematic representation of the performance-enhancement mechanisms of PIB5Cu. Enhancement mechanisms of (i) thermodynamic stability, (ii) kinetic activity and (iii) external field responsiveness. (b) Schematic representation of the formation of PIB5Cu and PIB, and explanation of the mechanism of the polarized H bond. (c) Higher H-bond energy, (d) lower dissociation barrier and (e) higher photothermal healing efficiency in PIB5Cu compared with PIB.

Here, we present a molecular design strategy of a Cu–*p*-benzoquinone dioxime–carbamate unit (Cu–BQDU) to address the ternary paradox of thermodynamics–kinetics–external field regulation (Fig. 1). Introducing Cu(II) to form coordination bonds in the system offers multiple advantages, as follows. (i) The strong positive electric field of Cu(II) attracts the electron cloud of carbonyl oxygen through coordination bonds, reducing the electron density of the adjacent secondary amine nitrogen via the inductive effect. This notably enhances the polarity of the N–H bond, boosting the hydrogen-bond (H-bond) strength in the carbamate–carbamate segments to the highest value (∼5.6 kcal/mol). (ii) The positive charge of Cu(II) induces a shift in the electron cloud of the N atom in the oxime group through coordination bonds, thereby enhancing its electron-accepting ability and significantly strengthening the π–π interactions. (iii) The superb multivalent synergy between coordination bonds, polarized hydrogen bonds and π–π interactions notably enhances the overall bond energy of Cu–BQDU, enabling the material to achieve the highest toughness among intrinsic photothermal elastomers (236.0 MJ/m^3^). This synergy also endows the material with excellent thermodynamic stability and notably prolongs its characteristic relaxation time at high temperatures. (iv) As a Lewis acid, Cu(II) catalyses the dynamic exchange of oxime–carbamate bonds. (v) The coordination bond extends the molecular conjugation length and optimizes the geometric matching of the π–π interaction, thereby enhancing the photothermal conversion efficiency. The latter two effects synergistically improve the external field and kinetic responsiveness of the material.

## RESULTS AND DISCUSSION

### Design and synthesis of polyurethane elastomer and PIB*x*Cu

The polyurethane elastomer was synthesized by using polytetramethylene ether glycol (PTMG), isophorone diisocyanate (IPDI), 1,4-benzoquinone dioxime (BQDO) and 2-ethyl-2-(hydroxymethyl)-1,3-propanediol (TMP) as starting materials. Dibutyltin dilaurate served as the catalyst and *N*, *N*-dimethylformamide (DMF) acted as the solvent to facilitate the reaction. CuCl_2_ was introduced into the polyurethane elastomers to form coordination bonds with oxime nitrogen and carbonyl oxygen (Fig. S1 and Table S1). Then, by adjusting the feeding ratio of CuCl_2_ and BQDO, a series of polyurethane elastomers were prepared. These elastomers were named PIB and PIB*x*Cu (where *x* = 2.5, 5, 7.5, 10). The coupling quadruple dynamic bonds (coordination bonds, H bonds, π–π interaction and oxime–carbamate bonds) built an ultra-strong Cu–BQDU with high comprehensive bond energy and high reversibility simultaneously. Cu(II) notably enhanced the overall bond energy of the locking units through H-bond polarization and optimizing the π–π interaction, endowing units with thermal stability and solvent resistance. At the same time, Cu(Ⅱ) acted as a Lewis acid to improve the reversibility of the oxime–carbamate bonds, synergistically enhancing the self-healing efficiency when coupled with improved photothermal performance.

### Toughening mechanism and effect of PIB and PIB*x*Cu (*x* = 2.5, 5, 7.5, 10)

To verify the successful synthesis of PIB and PIB*x*Cu, attenuated total reflectance Fourier transform infrared spectroscopy (ATR–FTIR) was employed to confirm the chemical structures of the synthesized materials. Taking PIB and PIB5Cu as examples, as shown in Fig. S2, the peaks ranging from 2775 to 3000 cm^−1^ correspond to C–H, which were widely distributed in the PTMG, TMP and IPDI. Also, there were no peaks around 2265 cm^−1^, corresponding to isocyanate groups, indicating that all the isocyanate groups had completely reacted with the PTMG, BQDO and TMP. Furthermore, the absorption peaks at 1720, 1230 and 1180 cm^−1^ corresponded to the C=O, C–N and C–O stretching vibrations, respectively, which could verify the formation of oxime–urethane groups. The spectra of PIB and PIB5Cu both had a wide peak at 3328 cm^−1^, which indicated the existence of a large number of H-bonded N–H groups. It also could be observed that the peak of N–O blueshifted from 938 to 958 cm⁻^1^ and the intensity of the stretching peak weakened, which could confirm the coordination with N atoms [23]. X-ray photoelectron spectroscopy was employed to clarify the coordination mode of Cu(Ⅱ) (Fig. S3). The O 1s peak attributed to the carbonyl group shifted from 531.40 to 531.71 eV, while the N 1s peak significantly shifted from 398.82 to 399.75 eV. These results indicated a decrease in the electron density of the oxygen and nitrogen atoms, which might be attributed to the coordination between the Cu(Ⅱ) and O/N atoms. Overall, the above results proved the successful synthesis of PIB and PIB*x*Cu.

**Figure 2.**
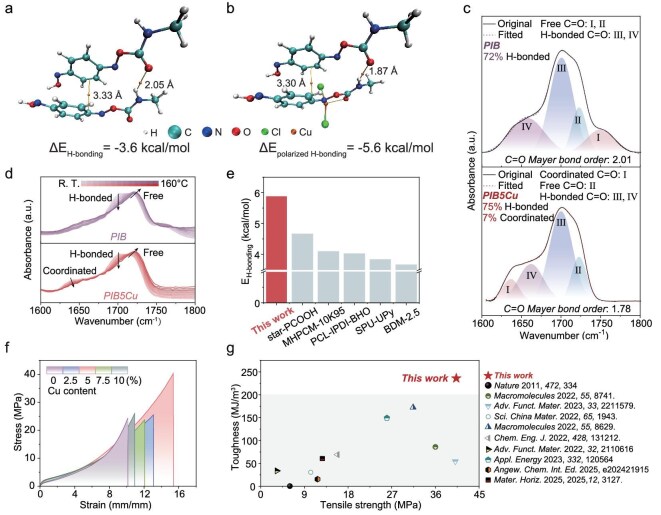
Toughening effect and mechanism of PIB and PIB*x*Cu (*x* = 2.5, 5, 7.5, 10). (a) Atoms-in-molecules topology analysis map of the BQDU–BQDU and (b) Cu–BQDU–BQDU dimers, derived from DFT calculations. Bond path and bond critical point are highlighted by the orange lines and ball. The H-bond length is marked. The gray, cyan, blue, red, orange and green balls represent the hydrogen, carbon, nitrogen, oxygen, copper and chlorine atoms, respectively. (c) FTIR spectra of the PIB and PIB5Cu elastomers in the C=O stretching vibration region. (d) Temperature-dependent FTIR spectra of the PIB and PIB5Cu elastomers in the C=O stretching vibration region, measured upon heating from room temperature to 160°C. (e) Comparison of H-bond-binding energy in different model compounds [34–38]. (f) Stress–strain curves of PIB and PIB*x*Cu (*x* = 2.5, 5, 7.5, 10) recorded at a deformation rate of 50 mm/min. (g) Ashby plot of toughness versus tensile stress for intrinsic photohealable elastomers.

We performed density functional theory (DFT) calculations [24] and independent gradient models based on the Hirshfeld partition (IGMH) [25] to determine and visualize the binding energies of the Cu–BQDU–BQDU (CuB–B) dimers with coordination bonds and BQDU–BQDU (B–B) dimers without coordination bonds. The optimized structural models of the dimers indicated that the H bonds and π–π interaction were the primary forces driving the interaction between the B–B/CuB–B segments. The binding energy of the B–B dimer (Δ*E* = −20.2 kcal·mol⁻^1^) was lower than that of the CuB–B dimer (Δ*E* = −26.8 kcal·mol⁻^1^) (Fig. S4). Furthermore, the distances of the H bonds and π–π interaction in CuB–B (1.87 and 3.30 Å) were much shorter than those in B–B (2.05 and 3.33 Å), indicating that the formation of the coordination bond improved the strength of the H bonds and π–π interaction, thereby bringing the polymer chains closer (Fig. 2a and b). To further validate the improved H-bonding interactions, peak deconvolution analysis was performed on the C=O stretching region in the FTIR spectra of the PIB and PIB5Cu elastomers (Fig. 2c) and temperature-dependent FTIR was employed to assign the identities of free and associated C=O groups (Fig. 2d). The C=O stretching region was deconvoluted into four subpeaks, corresponding to free (1750 and 1723 cm⁻^1^), disordered (1655 cm⁻^1^) and ordered (1700 cm⁻^1^) hydrogen bonds in the polyurethane groups. The results showed that the fraction of C=O groups involved in the H bonds was 72% for PIB and 75% for PIB5Cu, demonstrating that coordination strengthened the H-bond strength and promoted higher-density H-bond formation. The redshift of Peak I in the infrared spectrum (from 1750 to 1636 cm⁻^1^) was attributed to the partial transfer of electron density from the oxygen to the metal atom, which reduced the electron density of the C=O bond and consequently decreased its force constant. This observation was supported by the Mayer bond order [26], in which the bond order of the carbonyl group decreased from 2.01 to 1.78, indicating a reduction in the covalent character [27]. Combined with the DFT results, these findings confirmed that the polarized H bonds in PIB5Cu effectively promoted the formation of higher-density, shorter-distance and stronger H bonds compared with those in PIB [28]. The atoms-in-molecules theory was utilized for the properties at the bond critical point of the H bonds to characterize their features, enabling the simple and reliable calculation of the H-bond energies [29]. The results demonstrated that the H-bond-binding energy between the B–B units was at a relatively low value of 3.6 kcal/mol. In contrast, the CuB–B exhibited a much higher H-bond-binding energy of 5.6 kcal/mol—the highest reported to date for carbamate–carbamate H bonding in polyurethanes (Fig. 2e).

The π–π interactions were investigated by using IGMH because the area and volume of its isosurface exhibited a strong positive correlation with the interaction strength under certain conditions [30]. The results showed that, when the isosurface of the inter-fragment interaction function was defined at 0.002, the volume and area representing the π–π interactions increased from 4.5 Å^3^ and 39.4 Å^2^ for B–B to 4.8 Å^3^ and 41.9 Å^2^ for Cu–B–B (Figs S5 and S6). This might be attributed to a Cu-coordination-induced conjugation extension. Specifically, coordination extended the molecular conjugation length, promoted planarization of the conformation and facilitated intermolecular π–π interactions. The localized orbital locator [31] isosurface maps were generated for the two dimers to visualize the π-electron regions (Figs S7 and S8). It could be observed that the originally relatively independent conjugated pathways exhibited a certain degree of connectivity in the CuB–B dimer, which confirmed the extension of the conjugation length. An energy-decomposition analysis based on the force-field (EDA-FF) method [32] was used to decompose the total interaction energy between fragments into physically meaningful terms to investigate the nature of the interactions. In the π–π interactions, dispersion attractive forces drove the intermolecular binding, while electrostatic interactions further influenced the geometric structure [31]. This was confirmed by the EDA, which showed that the contribution of dispersion forces here was greater than that of the electrostatic interactions. The inter-fragment π–π interactions could be approximately estimated by subtracting the exchange repulsion term from the dispersion term. The dispersion interaction energy between CuB–B (4.6 kcal/mol) was significantly higher than that between B–B (3.0 kcal/mol), further verifying the enhancement of the π–π interaction strength (Tables S2 and S3).

As shown in Fig. S9, due to their robust cross-linked networks, PIB and PIB*x*Cu (*x* = 2.5, 5, 7.5, 10) exhibited excellent solvent resistance and could maintain their intact structures in DMF for >30 days. Fig. S10 shows that the swelling ratios of all the PIB*x*Cu samples were lower than those of PIB. Given the consistent chemical cross-linking density, the variation in swelling ratios might have arisen from strengthened physical cross-linking via coordination, polarized H bonds and optimized π–π interaction, which exhibited characteristics analogous to those of covalent cross-linking [33].

Tensile tests were conducted to evaluate the mechanical properties of PIB and PIB*x*Cu (*x* = 2.5, 5, 7.5, 10) (Fig. 2f and Fig. S11). In PIB*x*Cu with different Cu(II) molar ratios, as the molar fraction of Cu(II) increased from 2.5% to 10%, the tensile strength, toughness and Young’s modulus all exhibited a trend of increasing first and then decreasing. Notably, when the Cu(II) molar ratio reached 5%, PIB5Cu displayed optimal mechanical properties: the highest tensile strength (40.6 ± 1.8 MPa), maximum elongation (1584.6 ± 148.9%) and greatest toughness (236.0 ± 17.5 MJ/m^3^). These values notably exceeded those of PIB (tensile strength: 22.9 ± 1.9 MPa; elongation: 1033.1 ± 27.4%; toughness: 89.9 ± 14.1 MJ/m^3^; Young’s modulus: 5.6 ± 0.7 MPa) and other PIB*x*Cu variants. Moreover, the toughness of PIB5Cu surpassed those of all previously reported intrinsic photohealable elastomers (Fig. 2g).

Subsequently, cyclic stress–strain tests were performed on the three samples within a maximum strain range of 100%–500% (Figs S12 and S13). All three networks exhibited pronounced hysteresis, which could be primarily attributed to the dissociation of numerous H bonds within the polymer chains. However, coordination bonds, polarized H bonds and optimized π–π interactions also played important roles, as indicated by the quantitative analysis of energy dissipation for the three samples. The damping capacity, defined as the ratio of energy dissipation to incoming energy, was calculated by using cyclic tensile tests and the results are summarized in Fig. S14. Given their consistent covalent cross-linking network composition, variations in the damping capacity were attributed to the number and strength of the non-covalent interactions. The results demonstrated that, among the three networks tested, PIB5Cu exhibited the highest energy dissipation throughout, attributable to its highest density and strongest non-covalent interactions. Additionally, the shapes of the curves provided more information about the three networks. The curves for PIB2.5Cu and PIB5Cu declined with increasing strain, whereas the PIB curve declined initially and then increased. This phenomenon might have arisen because fewer non-covalent interactions forced covalent cross links to sustain the network integrity via elastic deformation under high strain.

The above speculations were further validated via cyclic stress–strain tests at a fixed strain of 100% to examine the recovery properties of the networks. Compared with PIB (1.2 MJ/m^3^), PIB5Cu exhibited a larger hysteresis loop area (1.7 MJ/m^3^), further corroborating the stronger non-covalent interactions in PIB5Cu (Fig. S15). After a rest at room temperature for 2 h before the sixth tensile cycle, the cyclic tensile curve of PIB5Cu was found to nearly completely overlap with that of the first cycle, whereas PIB showed a substantial decrease in stress. This observation aligned with the design expectation that the directed recombination of the high-bond-energy Cu–BQDU locking units accelerates segmental rearrangement within the polymer network, thereby enabling the material to recover its original shape and properties (Figs S16 and S17).

### Mechanism and performance of improved stability in PIB and PIB*x*Cu (*x* = 2.5, 5, 7.5, 10)

**Figure 3.**
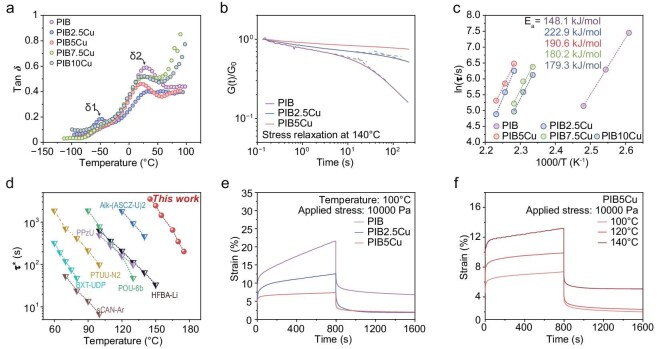
Thermal mechanical properties of PIB and PIB*x*Cu (*x* = 2.5, 5, 7.5, 10). (a) Tan *δ* of PIB and PIB*x*Cu (*x* = 2.5, 5, 7.5, 10) using DMA. δ_1_ and δ_2_ represent the glass transition temperatures (*T*_g_) of the soft segments and hard segments of the polyurethane, respectively. (b) Normalized stress relaxation curves of PIB and PIB*x*Cu (*x* = 2.5, 5) were measured at 140°C, respectively. (c) Activation energies were calculated at varying temperatures according to the Arrhenius equation, thereby characterizing their respective relaxation times. (d) Comparison of the characteristic relaxation time (τ*) of PIB5Cu with various covalent adaptive networks [39–45]. (e) Creep–recovery curves of PIB and PIB*x*Cu (*x* = 2.5, 5) with the same stress at 100°C. (f) Creep–recovery curves of PIB5Cu with the same stress level of 10 000 Pa at 100°C, 120°C and 140°C.

The findings above were due to the toughening mechanism and effects of Cu(Ⅱ) coordination. To further clarify how coordination cross-linking within the network influences mechanical performance, we conducted a series of measurements of the thermal mechanical properties. The thermogravimetric analysis curves revealed that the thermal decomposition temperature of PIB*x*Cu (*x* = 2.5, 5, 7.5, 10) all exceeded 294°C (Fig. S18). In the dynamic thermodynamic analysis (DMA) curve, δ_1_ and δ_2_ represented the glass transition temperatures (*T*_g_) of the soft segments and hard segments of the polyurethane, respectively. As shown in Fig. 3a, the observed peak at approximately –50°C was attributed to the movement of the PTMG soft segments, which was independent of the content of the hard segments. Given the consistent content of the hard segments, even though strong physical cross-linking composed of coordination bonds, polarized H bonds and optimized π–π interactions was formed in the hard segments, it had little effect on the mobility of both the polymer chain in the elastomer (Fig. S19). To further understand the temperature dependence of the viscoelasticity, the storage modulus (*G'*) and loss modulus (*G''*) were investigated in an extended temperature sweep and are plotted in Fig. S20. Both *G'* and *G''* of all three samples decreased with increasing temperature. The storage modulus consistently exceeded the loss modulus, indicating a predominantly elastic response and solid-like behavior during mechanical deformation.

Stress relaxation and creep experiments could also yield valuable structural information regarding the network. As shown in Fig. 3b, the stress applied to PIB released sharply and it almost completely relaxed in ∼40 s at 140°C. There was no obvious change in the relaxation behavior of PIB2.5Cu and PIB5Cu, in which considerable stress remained. Given that the relaxation behavior of the polymer networks was dependent on the cross links, these results further demonstrated that the dynamic cross-linking strength of PIB5Cu exceeded those of PIB and PIB2.5Cu. Traditional 1/e analysis of the stress relaxation experiments was used to determine the relaxation time (*τ*) when the normalized stress decreased to 36.8% of its initial value. A pronounced linear relationship was observed between ln(*τ*) and 1000/*T* (Fig. 3c and Fig. S21), with the slopes yielding apparent activation energies of 148.1, 222.9, 190.6, 180.2 and 179.3 kJ/mol for PIB, PIB2.5Cu, PIB5Cu, PIB7.5Cu and PIB10Cu, respectively. As this activation energy correlated with the dissociative capacity of dynamic cross links in the network, the much higher values for PIB*x*Cu (*x* = 2.5, 5, 7.5, 10) compared with those for PIB underscored their enhanced physical cross-linking stability. Additionally, the lower activation energy of PIB*x*Cu (*x* = 5, 7.5, 10) relative to that of PIB2.5Cu might be attributed to copper ions facilitating bond exchange within the network, thereby reducing the energy barrier for dynamic reconfiguration. Bond dissociation in the network resulted in stress relaxation and the scaling relationship of the characteristic relaxation times (τ*) with temperature was an important aspect of describing their rheological behavior. Notably, compared with many of the previously reported dynamic cross-linked polymers by different dynamic bonds, PIB5Cu exhibits longer τ* at a given temperature (Fig. 3d), suggesting that PIB5Cu possesses higher thermal stability compared with those polymers.

To further validate the thermal stability of the polymers, creep and recovery experiments were conducted on PIB, PIB2.5Cu and PIB5Cu under a constant stress of 10 000 Pa at 100°C. PIB, lacking Cu–BQDU, exhibited poor creep resistance at this temperature, with the strain exceeding 20% within 800 s. In contrast, PIB5Cu showed much lower strain than PIB over the same 800-s interval and its strain nearly fully recovered within 100 s after stress removal. Additionally, during recovery testing, both PIB2.5Cu and PIB5Cu displayed lower residual strain and higher recovery rates compared with PIB—attributes attributed to their higher physical cross-link density and strength. Remarkably, PIB5Cu rapidly achieved creep equilibrium (13.2%) at 140°C, exhibiting only 4% residual strain. This exceptional creep resistance was attributed to the high bond energy of the Cu–BQDU locking units within the network, which endows the material with thermodynamic stability under prolonged stress.

### The kinetic activity of PIB and PIB*x*Cu (*x* = 2.5, 5, 7.5, 10)

**Figure 4.**
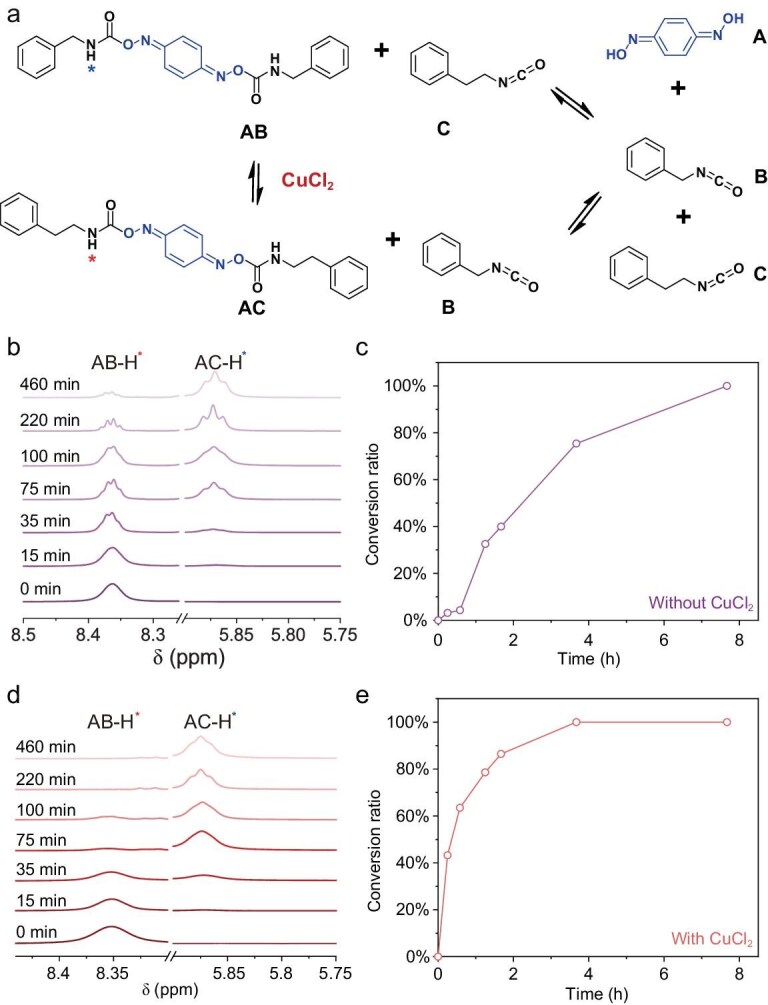
Model study of coupled multiple dynamic bonds. (a) Exchange reaction between AB and C produces AC and B. (b) ^1^H NMR spectra after mixing AB and C for different durations at 100°C. Proton peak intensities of AB gradually decreased over time, while those of AC gradually increased until an equilibrium was reached at ∼460 min. (c) Conversion of AB on reaching equilibrium without CuCl_2_ (conversion ratio (AB_0_–AB_t_)/(AB_0_–AB_eq_), AB_0_: initial concentration of AB, AB_t_: concentration of AB at time *t*, AB_eq_: equilibrium concentration of AB). (d) ^1^H NMR spectra after mixing AB, C and CuCl_2_ for different durations at 100°C. (e) Conversion of AB on reaching equilibrium with CuCl_2_.

After the thermodynamic stability validated our design, the corresponding experiments in this section confirmed the enhancement of its kinetic activity. To investigate the reversibility of the BQDU bonds and demonstrate the catalytic role of CuCl_2_, first, we synthesized compound AB—a small-molecule model containing oxime–carbamate bonds based on BQDO and benzyl isocyanate—and mixed it with phenethyl isocyanate (compound C) in deuterated dimethyl sulfoxide (Fig. 4a and Figs S22–S26). Then, *in situ*  ^1^H nuclear magnetic resonance (^1^H NMR) was used to monitor the reaction of compounds AB and C to AC and B at 100°C (Fig. 4b). The signals at 8.37 ppm in the ^1^H NMR spectra corresponded to the protons (marked as AB–H*) connected to the nitrogen atom (Fig. S27). As time went by, a new proton signal at 5.87 ppm corresponded to the proton (marked as AC–H*) connected to the nitrogen atom in the products (compound AC), indicating the occurrence of an exchange reaction (Fig. S28). It still had not reached equilibrium after being maintained at 100°C for >8 h (Fig. 4c). The *in situ*  ^1^H NMR spectra of compound AB were used to reveal its dissociation reaction (Figs S29 and S30), which showed that the dynamic exchange reaction of the oxime–carbamate bonds based on BQDO and benzyl isocyanate involves two steps. Firstly, the oxime–carbamate bonds in compound AB dissociated into BQDO groups in compound A and benzyl isocyanate in compound B. Compound A reacted with the isocyanate groups in compound C. Accordingly, the BQDU bonds had the property of dynamic reconstruction. Then, we added some CuCl_2_ into the aforementioned exchange reaction (Fig. 4d). Notably, the exchange reaction only took 4 h to reach equilibrium (Fig. 4e)—much sooner than that in the aforementioned exchange reaction without CuCl_2_. Hence, Cu(Ⅱ) indeed promoted the reversibility of the oxime–carbamate bonds based on the BQDO and isocyanate groups.

To further elucidate the role of CuCl_2_ in the dynamic dissociation and association of BQDU bonds at both ends, we employed DFT calculations by using BQDO and methyl isocyanate as the model systems to probe the catalytic effect of CuCl_2_ (red line) compared with the case in which no CuCl_2_ was used (black line) in a two-step concerted reaction (Fig. S31). The subsequent dissociation of the destabilized oxime–carbamate bond of INT1 was located as TS1, in which the breaking of the C=O bond was accompanied by the proton migration from N to O to produce INT2 and isocyanate. The predicted free-energy barrier was 34.1 kcal mol^−1^, which was slightly lower than that without the catalyst. Further fragmentation of INT2 to yield FS1 was also investigated. Computational results implied that the Δ*G* required for further fragmentation (35.5 kcal/mol) was 14.2 kcal mol^−1^, which was much lower than that for INT5 to yield FS2 (49.7 kcal/mol). The result demonstrated that such interactions exert a catalytic effect on the dissociation of both ends of the oxime–carbamate moiety. This also confirmed that the presence of copper coordination enhances its kinetic responsiveness.

### External field responsiveness of PIB and PIB*x*Cu (*x* = 2.5, 5, 7.5, 10)

**Figure 5.**
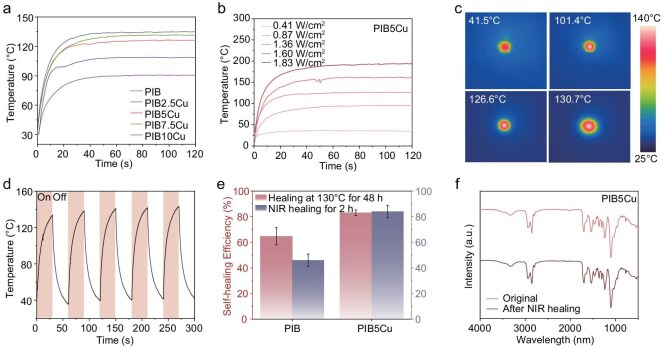
Photothermal effect and photo-healing properties of PIB and PIB*x*Cu (*x* = 2.5, 5, 7.5, 10). (a) Heating temperature curves of PIB and PIB*x*Cu (*x* = 2.5, 5, 7.5, 10) under 1.36 W/cm^2^ 808-nm NIR laser. (b) Surface temperature profile of PIB5Cu upon exposure to 808-nm NIR laser. (c) Infrared image of PIB5Cu recorded by using an infrared camera at different irradiation times. (d) Heating temperature curves of PIB5Cu under irradiation of an 808-nm NIR laser with different powers. (e) Maximum surface temperature of PIB5Cu changes under switching NIR irradiation of 1.36 W/cm^2^ for five cycles. (f) Comparison of self-healing efficiency between PIB and PIB5Cu healing at 130°C for 48 h and NIR for 2 h.

Previous studies have confirmed that Cu–BQDU critically enhances both thermodynamic stability and kinetic activity in the polymer network. As is well known, materials with a black appearance can absorb a wide range of wavelengths of light to generate heat. The reaction between BQDO and isocyanates further increases the grayscale. This is because carbamate, as an electron-withdrawing group, can extend the conjugated structure and shift it to longer wavelengths [46]. The coordination of copper may further enhance this effect. To further validate the capacity of the material for external field responsiveness, this chapter systematically investigated its photothermal conversion efficiency and underlying enhancement mechanisms. Specifically, temperature variations in PIB and PIB*x*Cu (*x* = 2.5, 5, 7.5, 10) under fixed laser irradiation power were monitored to quantitatively compare their photothermal performance. As shown in Fig. 5a, when the power density of the 808-nm near-infrared (NIR) light was adjusted to 1.36 W/cm² and the samples of PIB and PIB*x*Cu materials were irradiated respectively at a room temperature of 25°C, the samples exhibited a strong photothermal effect. The surface temperatures of the materials increased from room temperature to 90.6°C, 108.7°C, 126.4°C, 131.5°C and 135°C, respectively (Fig. S32). This indicated that the content of Cu(II) effectively enhanced the photothermal conversion efficiency of the polymer, which might be attributed to optimized π–π interactions enhancing NIR absorption and leading to more light energy being captured. The influence of the irradiation intensity on the photothermal effect was further explored. For the same irradiation time, as the irradiation intensity was increased, when the power densities were set at 0.41, 0.87, 1.36, 1.6 and 1.83 W/cm², respectively, the highest stable temperatures within 120 s were 34.6°C, 94.1°C, 130.5°C, 161.7°C and 192.9°C, respectively (Fig. 5b). Therefore, the temperature of the samples can be flexibly controlled by adjusting the laser irradiation intensity. The thermal images captured under NIR light radiation clearly show the temperature changes in the irradiated area, with the temperature gradually spreading outward from the irradiated site (Fig. 5c). To evaluate the photothermal stability, PIB5Cu was irradiated with 1.36 W/cm^2^ of NIR light for 30 s, followed by cooling to ambient temperature, and this cycle was repeated five times. The results showed that the surface temperature of the sample increased slightly after the five cycles, while the photothermal conversion curves showed no significant change and this result confirmed the excellent photothermal stability of the material (Fig. 5d). Collectively, PIB5Cu exhibits robust and efficient photothermal conversion performance under NIR laser irradiation. We systematically evaluated the self-healing performance of PIB5Cu and PIB. As recorded by using optical microscopy, the scratch on the PIB5Cu film could be repaired after 1 h under NIR (Fig. S33). Dumbbell-shaped specimens of PIB5Cu and PIB were each completely cut into two segments and subjected to NIR irradiation. Benefitting from its enhanced photothermal effect, the surface temperature of PIB5Cu rose to nearly 130°C—much higher than that of PIB. After 2 h of irradiation, PIB5Cu exhibited a healing efficiency of 83.9% (33.7 ± 1.2 MPa), whereas PIB achieved only 43.5% (10.5 ± 1.3 MPa). Mechanistically, photothermal-based elastomer repair fundamentally relies on thermal action. When the PIB5Cu and PIB specimens were heated at 130°C for 48 h, the healing efficiency of PIB5Cu remained much higher than that of PIB, attributed to its higher kinetic activity and faster external field response rate (Fig. 5e). The material showed no obvious structural changes before and after the high-temperature healing, reflecting its stable thermodynamic properties (Fig. 5f). Notably, the healing efficiency of PIB5Cu after 48 h of high-temperature treatment was nearly identical to that after 2 h of NIR irradiation, highlighting the precision and efficiency of photothermal healing. Collectively, these experiments demonstrate that, by introducing Cu(Ⅱ)-coordination bonds, the PIB5Cu elastomer achieves the synergistic optimization of thermodynamic, kinetic and external field response properties.

## CONCLUSION

We have created the toughest photothermal self-healing elastomer via Cu(II)-coordinated dynamic cross-linking unit. By chemically coupling quadruple dynamic bonds, we have built a robust Cu–BQDU with simultaneously high overall bond energy and reversibility. Cu–BQDU achieves the synergistic enhancement of multiple performance metrics through two distinct yet interdependent mechanisms. First, the coordination bonds in Cu–BQDU not only reinforce the cross-linked network and polarize the H bonds (the strongest H bond in the reported carbamate–carbamate segment (∼5.6 kcal/mol)), but also optimize the π–π interactions to enhance the thermodynamic stability and toughness. Second, leveraging the Lewis acidity of Cu(II), the dynamic exchange of oxime–carbamate bonds is catalysed, endowing the material with adaptive kinetic properties. The optimized π–π interactions also boost the photothermal conversion efficiency (photothermal equilibrium temperature increased by 39.3%), creating a feedback loop that integrates stimulus responsiveness. Overall, this work proposes and realizes the strategy of simultaneously tuning thermodynamic, kinetic and external field responsiveness to modulate the properties of a material, representing a paradigm-shifting design principle with profound implications for developing next-generation materials. The power of this strategy has been demonstrated through an elegant molecular design to concurrently improve the mechanical properties, stability, photothermal conversion efficiency and self-healing efficiency of the mutually restrictive polymer.

## Supplementary Material

nwaf521_Supplemental_Files
