# Supplementary material for: Ultrastable, supertough and photohealable polymer
Source: Natl Sci Rev. 2025 Nov 27;13(1):nwaf521. doi: 10.1093/nsr/nwaf521 (PMC12796801; doi:10.1093/nsr/nwaf521)
Supplement: nwaf521_Supplemental_Files [file nwaf521_supplemental_files.zip › Supplementary data.pdf]

Supplementary data for

Ultrastable, supertough and photohealable polymer

Zekai Wu *et al.*

*Corresponding authors: Zhengwei You, zyou@dhu.edu.cn

This PDF file includes:

Supplementary Text
Figure S1 to S33
Table S1 to S3

Supplementary data

1. General methods

General Characterization Information: All tests were performed at room temperature unless otherwise noted. Nuclear magnetic resonance (NMR) spectra were recorded with a Bruker Avance 600 spectrophotometer with the use of the deuterated solvent as the lock and the residual solvent or TMS as the internal reference.

FTIR spectra were recorded on a Nicolet 8700 spectrometer (Thermo Electron Corporation, USA). Attenuated total reflectance Fourier transform infrared (ATR-FTIR) spectra were recorded on a ThermoFisher Scientific Nicolet iS50 spectrometer with an ATR accessory. To collect in-situ variable temperature FTIR spectra, the film sample was fitted to the stretching bench and then heated from room temperature to 160 °C. The absorption spectra were measured within the wavenumber region of 4000-400 cm^{-1} at a resolution of 4 cm^{-1} with 32 scans at a heating or cooling rate of 5 °C min^{-1}. Besides, the spectra at various temperatures were normalized by the stretching peaks of methylene.

After cutting the samples into rectangular specimens with dimensions of 2 mm × 5 mm × 0.8 mm and vacuum-drying them in an oven, immerse the specimens in a sample vial containing 5 mL of N, N-dimethylformamide (DMF) solvent. Remove the specimens every 30 minutes, blot off the excess solvent with lint-free paper, weigh them on an electronic balance, and calculate the swelling ratio of the specimens using the following equation:

$$S = \frac{M_t - M_0}{M_0} \times 100\%$$

The glass transition temperature is obtained by dynamic thermodynamic analysis on a DMA1 (METTLER TOLEDO). Dynamic mechanical analyzer. Rectangular samples (ca. 1 mm (T) × 5 mm (W) × 10 mm (L)) were tested at a frequency of 1 Hz and a strain of 0.1%. Heating ramps of 5 ℃ min^{-1} were applied from -100 to 100 °C. T_g values were calculated from the maximum value of tan Delta.

Thermogravimetric analysis (TGA) was used to study the dynamic thermal stability utilizing a thermogravimetric analyzer (Libra/209F1). The synthesized copolymers were evaluated to 600 °C using a heating rate of 10 °C·min^{-1} under a nitrogen atmosphere.

Temperature sweep tests were performed on an Anton Paar MCR702 rheometer equipped with an 8 mm solid-mode rotor. The tests applied a 0.5% strain at a frequency of 1 Hz to samples that were 1 mm thick. The normalized stress-relaxation experiments were performed in a strain control (10% strain) mode. The relaxation modulus (G) was normalized by the initial value (G_0). E_a represents the activation energy, τ_0 represents the characteristic relaxation time at 1/e, R is the gas constant, T is the temperature relaxation test, and τ(T) corresponds to the time when the modulus relaxes to 1/e. E_a can be calculated by multiplying the slope obtained by plotting ln(τ)-1000/T by the constant R. The characteristic relaxation time (τ*) was defined as the time required for G/G0 = 1/e with exponential decay function: $G(t) = G_0 \exp(-t/\tau^*)$.

The mechanical properties of the samples were assessed by an MTS E42 tensile

machine equipped with a 100 N load cell. The uniaxial tensile tests were carried out while the crosshead was adjusted at 50 mm·min^{-1}. Rectangular tensile bars (1 mm (T) × 3 mm (W) × 20 mm (L)) cut from large films were used. The deflection rate of uniaxial tensile measurements was 50 mm min^{-1}. At least three specimens were tested and averaged for each sample. If not specified, cycle tensile tests were performed at a tensile rate of 50 mm·min^{-1} and a recovery rate of 50 mm min^{-1}. Energy dissipation was calculated by integrating the area encompassed by the cyclic tensile curves. Damping capacity was defined as the ratio of the dissipated energy (the area encompassed by the loading and unloading curves) to the loading energy (the area encompassed by the loading curve).

Self-healing tests were evaluated mainly by scratch recovery and restoration of mechanical properties for various periods. The photothermal effect of PIB and PIBxCu (x=2.5, 5, 7.5, 10) was performed under 808 nm NIR laser (Shanghai Xilong Optoelectronic Technology Co., DL-808-2000-T2), and the temperature data were collected by FLIR (German Detu Instrument Co., TESTO-885-2). Prepare a dumbbell-shaped specimen. The specimen has a length of 35 mm, a width of 6 mm at both ends and 2 mm in the middle, and a thickness of 0.5 mm. Fix an 808 nm spatially output laser on an iron stand, positioning it 40 cm directly above the material.

Adjust the power density of the laser output light, monitor the actual temperature of the specimen's surface using an infrared thermal imager, and record the temperature change of the sample over time under the irradiation of 808 nm near-infrared (NIR) light. Completely cut the dumbbell-shaped specimen with a razor blade. Then, bring the two cross-sections of the specimen into contact and align them properly. Fix the 808 nm spatially output laser 40 cm directly above the specimen. Select NIR light at 808 nm with an appropriate power density to irradiate the cross-section of the specimen. After a certain period of irradiation, conduct a uniaxial tensile test on the healed specimen using an electronic universal testing machine to obtain the tensile strength of the material after photo-healing. We define the photo-healing efficiency of the material as the ratio of the tensile strength of the specimen after photothermal healing to the original tensile strength of the specimen. The photohealing performance of the material is evaluated by calculating the photohealing efficiency.

2. DFT calculations

Geometries of all dimers were fully optimized using the B3LYP functional[1, 2] with the ma-TZVP basis set[3, 4] and the empirical dispersion correction[5] within a Gaussian 16 package[6] (Gaussian 16, Revision A.01, M. J. Frisch, G. W. Trucks, H. B. Schlegel, G. E. Scuseria, M. A. Robb, J. R. Cheeseman, G. Scalmani, V. Barone, G. A. Petersson, H. Nakatsuji, X. Li, M. Caricato, A. V. Marenich, J. Bloino, B. G. Janesko, R. Gomperts, B. Mennucci, H. P. Hratchian, J. V. Ortiz, A. F. Izmaylov, J. L. Sonnenberg, D. Williams-Young, F. Ding, F. Lipparini, F. Egidi, J. Goings, B. Peng, A. Petrone, T. Henderson, D. Ranasinghe, V. G. Zakrzewski, J. Gao, N. Rega, G. Zheng, W. Liang, M. Hada, M. Ehara, K. Toyota, R. Fukuda, J. Hasegawa, M. Ishida, T. Nakajima, Y. Honda, O. Kitao, H. Nakai, T. Vreven, K. Throssell, J. A. Montgomery, Jr., J. E. Peralta, F. Ogliaro, M. J. Bearpark, J. J. Heyd, E. N. Brothers, K. N. Kudin, V. N. Staroverov, T.

A. Keith, R. Kobayashi, J. Normand, K. Raghavachari, A. P. Rendell, J. C. Burant, S. S. Iyengar, J. Tomasi, M. Cossi, J. M. Millam, M. Klene, C. Adamo, R. Cammi, J. W. Ochterski, R. L. Martin, K. Morokuma, O. Farkas, J. B. Foresman, and D. J. Fox, Gaussian, Inc., Wallingford CT, 2016.). Single-point energy calculations were performed for all stable conformations at the same level of theory to obtain the binding energies (ΔE). The ΔE values of dimers were evaluated by equations:

$$\Delta E = E_{AB} - E_A - E_B$$

E_{AB}, E_A, E_B represent the energy of complex AB, pure A, and pure B, respectively.

The PBE0-D3(BJ)[7] density functional method (DFT) was employed to carry out all the energy surface calculations. The nonmetallic atoms were described with the def-TZVP basis set[3], while the SDD basis set[8] was employed for copper atoms. Vibrational frequency analyses at the same level of the theory were performed on all the optimized geometries to characterize them as local minima (no imaginary frequency) or transition states (one imaginary frequency). In addition, intrinsic reaction coordinate[9] (IRC) calculations were used to verify that the transition state connects with the appropriate reactant and product. The gas-phase Gibbs free energies for all species were obtained at 298.15 K and 1 atm at their respective optimized structures. The def2-TZVP basis set[10] was utilized for single-point energy calculation. The Gibbs free energy was determined by adding the single-point energy and the gas-phase thermal correction to the Gibbs free energy obtained from the vibrational frequency analyses.

Atom in molecule (AIM) theory[11-13] was employed to gain an insight into H-bonding strengths in all H-bonded aggregates, thus evaluating the influence of hydrogen bonding on mechanical properties. Energy decomposition analysis based on forcefield, which can be abbreviated as EDA-FF[14], enables the dissection of the total interaction energy between fragments into physically meaningful energy terms. This approach facilitates the exploration of the nature of interactions. Moreover, the results are visualizable, allowing for convenient examination of weak intramolecular interactions. The Localized Orbital Locator (LOL)[15], a real-space function used to investigate electron delocalization pathways, was employed here to exclusively examine the characteristics of π-electron structures. Given Gaussian results, the independent gradient model based on Hirshfeld partition (IGMH)[16], AIM analysis, EDA-FF, and LOL were performed using the Multiwfn 3.9 programs[17, 18]. All the IGMH, AIM, and LOL-π isosurface maps were rendered by Visual Molecular Dynamics (VMD) 1.9.3 program[19]. Additionally, using the IGMH method to calculate the area of π-π isosurface has a positive correlation with interaction strength. Typically, electron density at the bond critical point (ρBCP) was used to quantitatively describe the property of hydrogen bonds. The formula for calculating the binding energy (ΔE) of hydrogen bonds is given below[20]:

$$\Delta E = -223.08 \times \rho(r_{BCP}) + 0.7423$$

According to the absolute value of hydrogen binding energy (|ΔE|), H-bonds could be divided into weak (|EHB| < 10.5 kJ mol^{-1}), weak to medium (10.5 kJ mol^{-1} < |EHB| < 46.0 kJ mol^{-1}), medium (46.0 kJ mol^{-1} < |EHB| < 62.8 kJ mol^{-1}), and strong (|EHB| > 62.8 kJ mol^{-1}).

3. Experimental

Materials

1,4-Benzoquinone dioxime (BQDO, 95%) was purchased from Adamas-beta. Copper chloride ($CuCl_2$, 99%) was purchased from Beijing Sinopharm Chemical Reagent (China). PTMG (M_n = ~1000 g mol^{-1}) and isophorone diisocyanate (IPDI, 99%) and dibutyltin dilaurate (DBTDL, 95%) were purchased from Aladdin. Trimethylolpropane (TMP, 98%) was purchased from Aladdin. Phenethyl isocyanate (98%) and benzyl isocyanate (98%) were purchased from Aladdin. N, N-dimethylformamide (DMF) were purchased from J&K Scientific (China).

Synthesis of PIB

The synthesis route of PIB was shown in scheme 1. PTMG (4 g, 4 mmol) was added to a glass reactor equipped with a magnetic stirred and dried at 110 °C for 2 h under vaccum. After cooling to 80 °C, IPDI (2.3651 g, 10.64 mmol) was added dropwise to the reactor and heated to 110 °C for 2 h under a nitrogen atmosphere. After cooling to 80 °C, 1,4-Benzoquinone dioxime (BQDO, 0.5525 g, 4 mmol), DBTDL (0.2wt%) and DMF solvent (9 ml) were added into the glass reactor and reacted for 19 h. Then, Trimethylolpropane (TMP, 0.2361 g, 1.76 mmol) and 2 ml DMF were added to react for 4 h. Finally, the solution was poured into a polyetrafluoroethylene mold and reacted for 48h for further curing in a vaccum from 30 °C to 80 °C to produce PIB film.

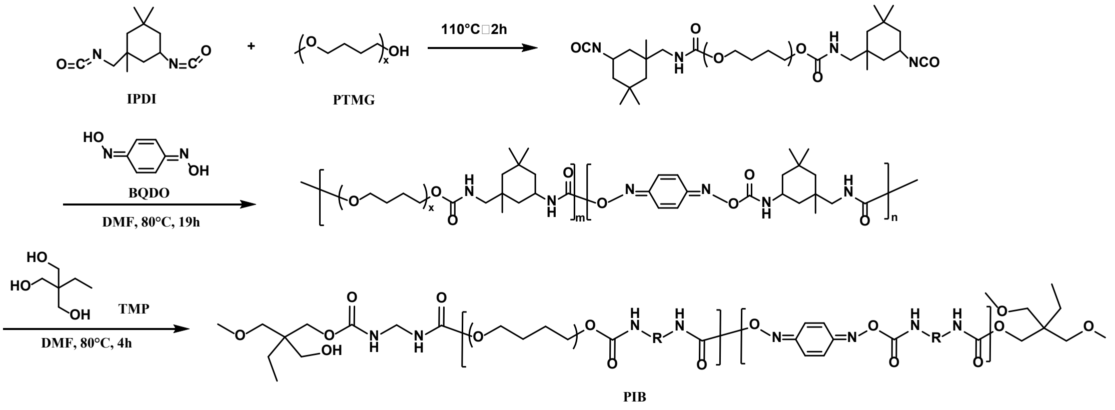

Figure S1 The synthesis route of PIB

Synthesis of PIBxCu (x=2.5, 5, 7.5, 10)

PTMG (4 g, 4 mmol) was added to a glass reactor equipped with a magnetic stirred and dried at 110 °C for 2 h under the vaccum. After cooling to 80 °C, IPDI (2.3651g, 10.64 mmol) was added dropwise to the reactor and heated to 110 °C for 2 h under a nitrogen atmosphere. After cooling to 80 °C, 1,4-Benzoquinone dioxime (BQDO, 0.5525 g, 4 mmol), DBTDL (0.2wt%), $CuCl_2$ and DMF solvent (9 ml) were added into the glass reactor and reacted for 19 h. Then, Trimethylolpropane (TMP, 0.2361 g, 1.76 mmol) and 2 ml DMF were added to react for 4 h. Finally, the solution was poured into a polyetrafluoroethylene mold and reacted for 48 h for further curing in a vaccum from 30 °C to 80 °C to produce PIBxCu film. To explore the effect of addition of $CuCl_2$, we designed 4 different formulations of PIB with different molar ratio of a ($CuCl_2$): b (BQDO) (a= 2.5%, 5%, 7.5%, 10%; b = 1) and the specific formulas were in Table S1.

Table S1The composition of all examples

Sample	PTMG-1000	IPDI	BQDO	TMP	DBTDL	$CuCl_2$
PIB	4mmol	10.64mmol	4mmol	1.76mmol	0.2wt%	——
PIB2.5Cu	4mmol	10.64mmol	4mmol	1.76mmol	0.2wt%	0.1mmol
PIB5Cu	4mmol	10.64mmol	4mmol	1.76mmol	0.2wt%	0.2mmol
PIB7.5Cu	4mmol	10.64mmol	4mmol	1.76mmol	0.2wt%	0.3mmol
PIB10Cu	4mmol	10.64mmol	4mmol	1.76mmol	0.2wt%	0.4mmol

4. Characterization for PIB and PIBxCu (x=2.5, 5, 7.5, 10)

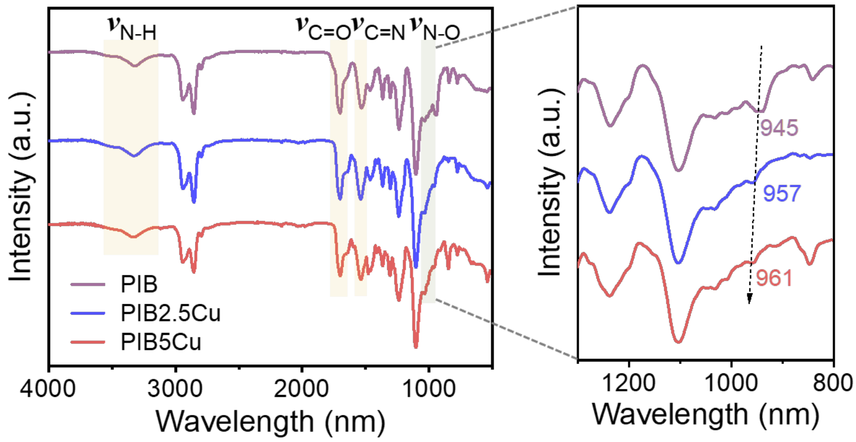

Figure S2 FTIR spectra of PIB, PIB2.5Cu, and PIB5Cu.

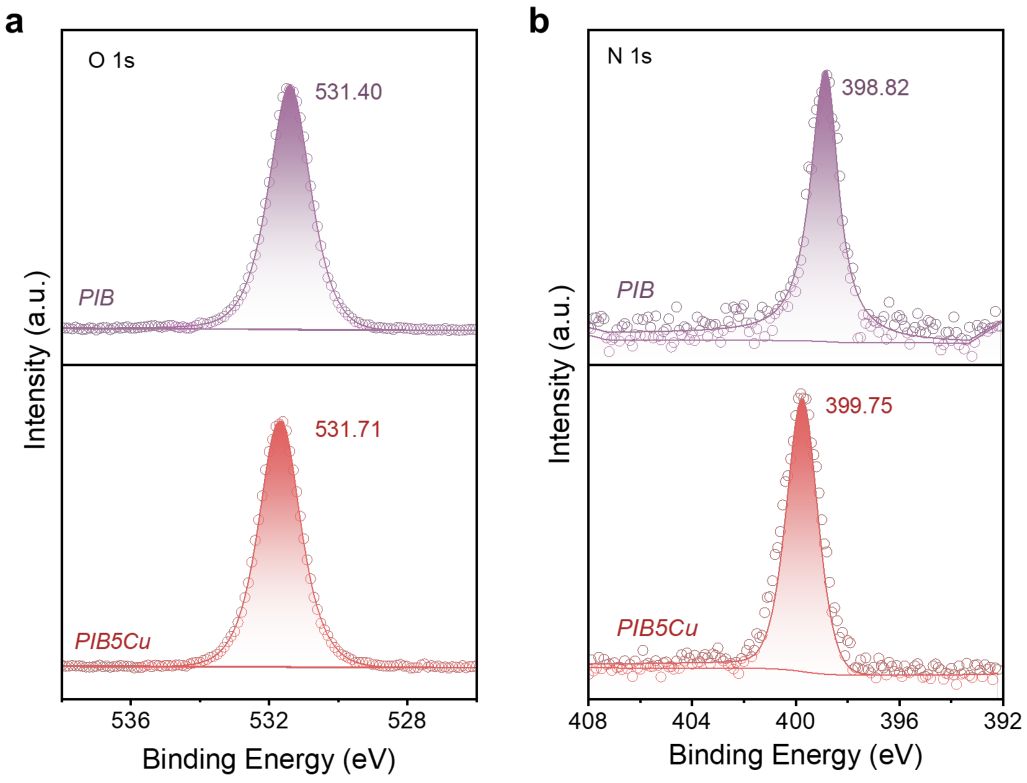

Figure S3 XPS spectra of PIB and PIB5Cu.

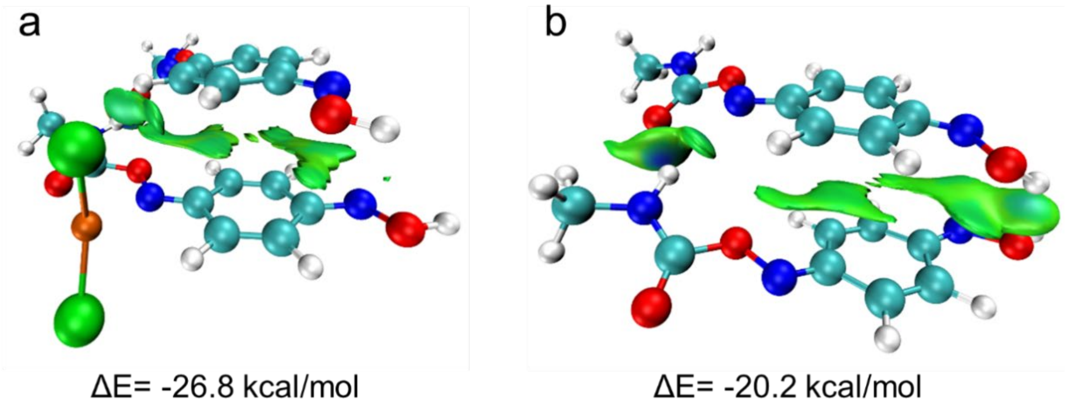

Figure S4 The intermolecular interaction of (a) Cu-BQDU-BQDU and (b) BQDU-BQDU dimer was investigated by independent gradient model (IGM).

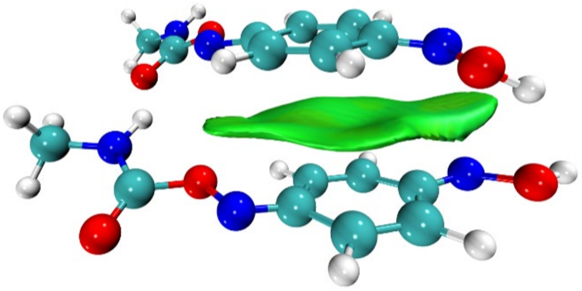

Figure S5 The π-π interaction of BQDU-BQDU (B-B) dimer was investigated by independent gradient model (IGM).

Volume: 30.49063 Bohr^3 (4.51825 Angstrom^3)

Estimated density according to mass and volume (M/V): 143.4613 g/cm^3

Overall surface area: 140.75865 Bohr^2 (39.41644 Angstrom^2)

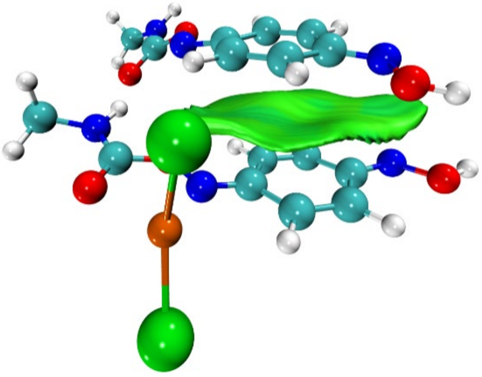

Figure S6 The π-π interaction of Cu-BQDU-BQDU (Cu-B-B) dimer was investigated by independent gradient model (IGM).

Volume: 32.40851 Bohr^3 (4.80245 Angstrom^3)

Estimated density according to mass and volume (M/V): 181.4609 g/cm^3

Overall surface area: 149.65392 Bohr^2 (41.90737 Angstrom^2)

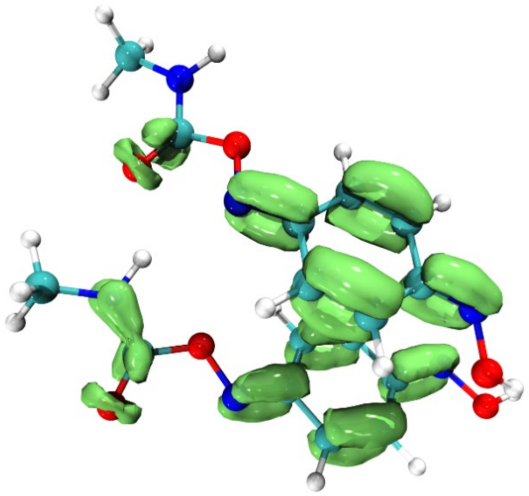

Figure S7 The LOL-π isosurface plot of BQDU-BQDU (B-B) dimer.

Table S2 Energy decomposition analysis based on forcefield of BQDU-BQDU (B-B) dimer.

	Electrostatic	Repulsion	Dispersion	Total
B-B	0.98 kJ/mol	31.29 kJ/mol	-46.87 kJ/mol	-14.61 kJ/mol

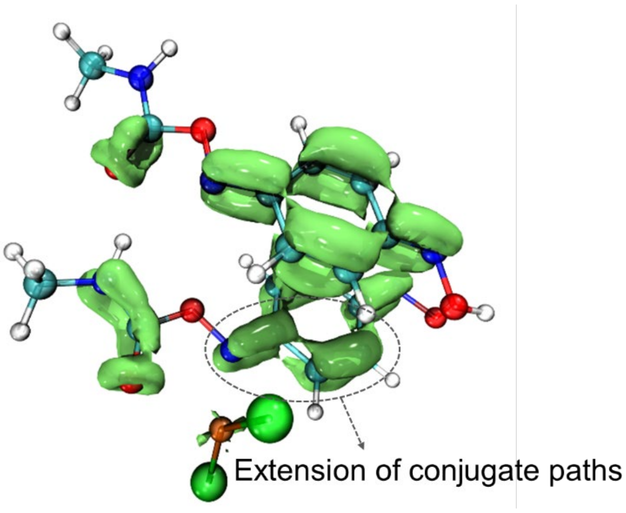

Figure S8 The LOL-π isosurface plot of Cu-BQDU-BQDU (CuB-B) dimer.

Table S3 Energy decomposition analysis based on forcefield of Cu-BQDU-BQDU (CuB-B) dimer

	Electrostatic	Repulsion	Dispersion	Total
CuB-B	-14.61 kJ/mol	34.67 kJ/mol	-53.92 kJ/mol	-34.62 kJ/mol

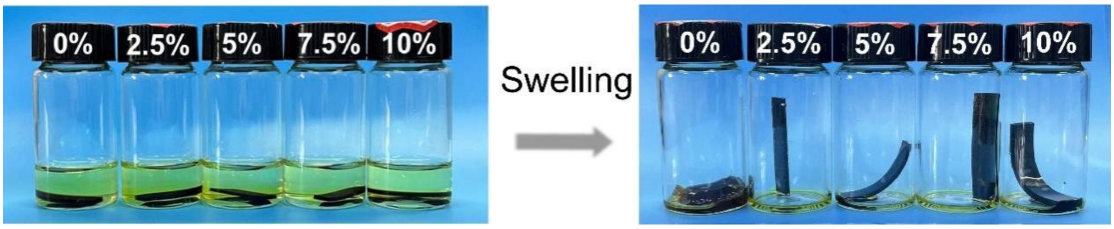

Figure S9 Photos of PIB and PIBxCu (x=2.5, 5, 7.5, 10) before and after swelling.

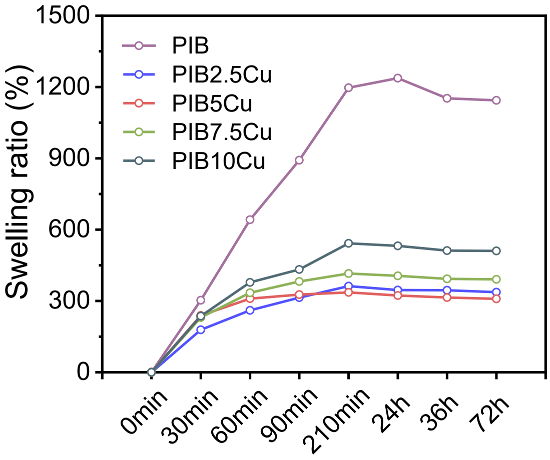

Figure S10 Swelling ratio curves of PIB and PIBxCu (x=2.5, 5, 7.5, 10).

5. Mechanical properties of PIB and PIBxCu (x=2.5, 5, 7.5, 10)

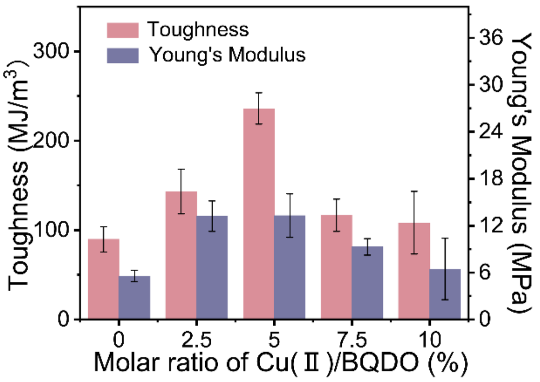

Figure S11 Toughness and Young's modulus of PIB, PIBxCu (x=2.5, 5, 7.5, 10) were calculated from their stress-strain curves.

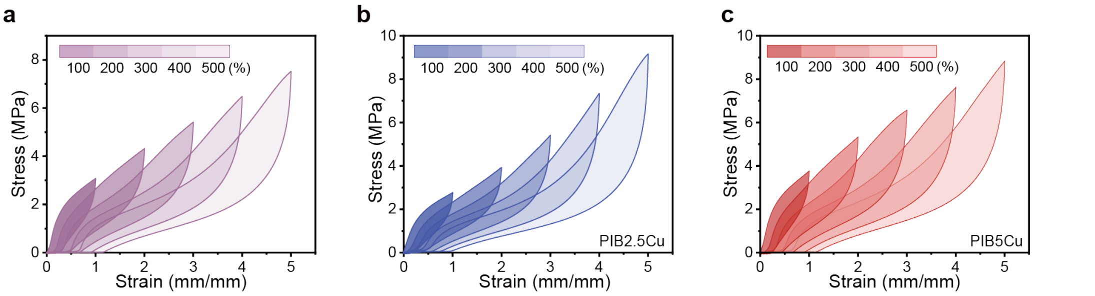

Figure S12 The cyclic tensile tests with gradually larger strains of (a) PIB, (b) PIB2.5Cu, and (c) PIB5Cu.

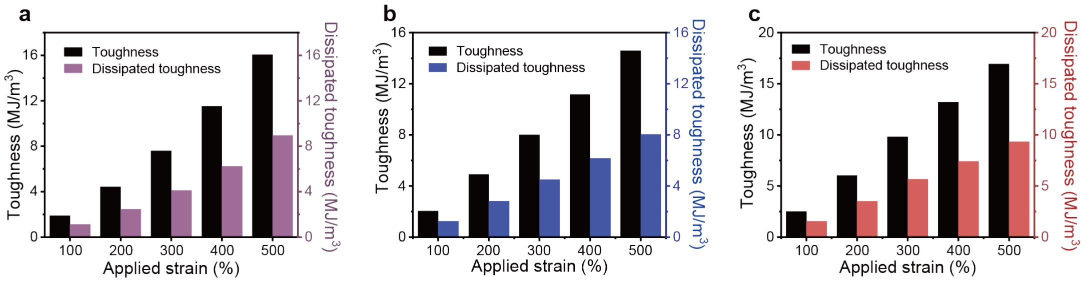

Figure S13 The total and dissipated toughness calculated for (a) PIB, (b) PIB2.5Cu, and (c) PIB5Cu within five cyclic stress-strain tests in the maximum strain range from 100% to 500%.

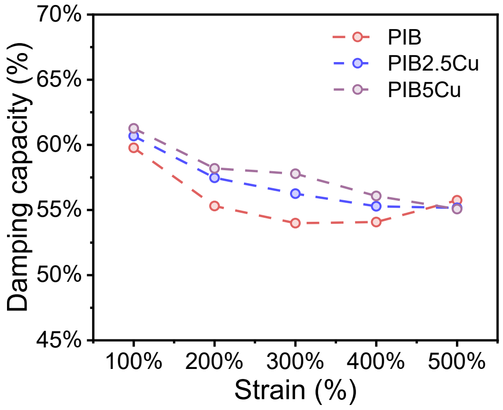

Figure S14 Damping capacities of PIB, PIB2.5Cu, and PIB5Cu calculated based on their cyclic tensile tests.

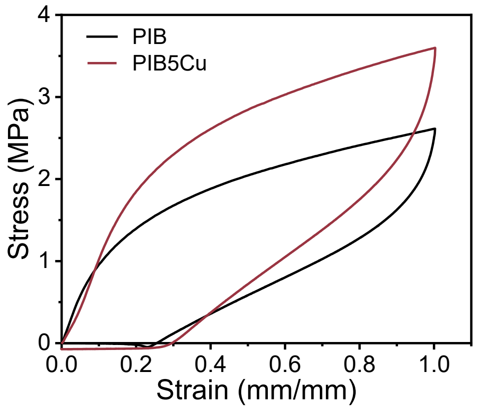

Figure S15 Cycle tensile test of PIB and PIB5Cu with an applied maximum strain of 100% for the first loading and unloading.

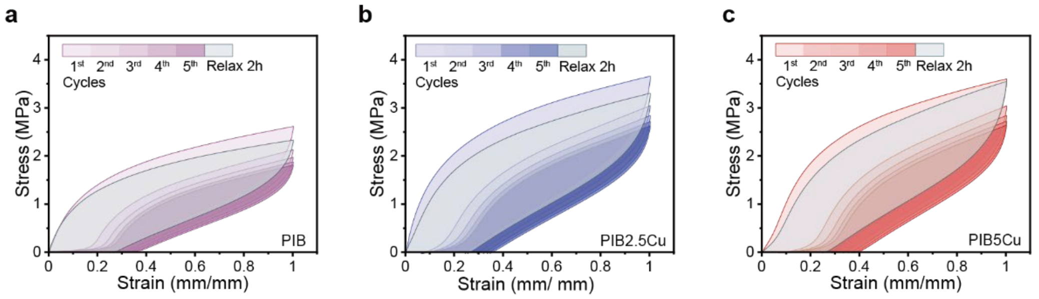

Figure S16 The cyclic tensile tests of (a) PIB, (b) PIB2.5Cu, and (c) PIB5Cu.

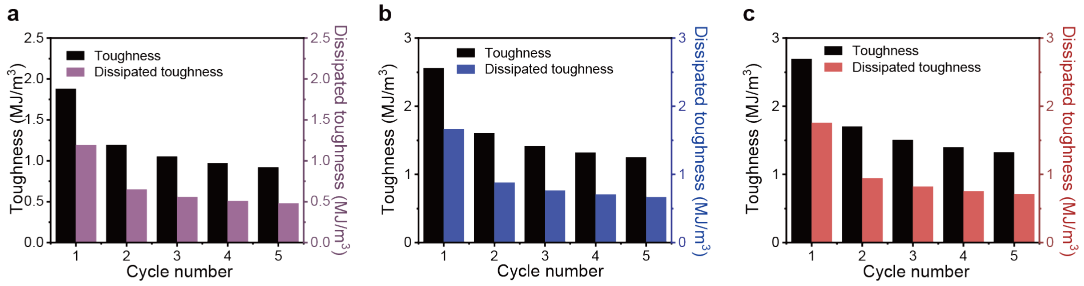

Figure S17 The total and dissipated toughness calculated for (a) PIB, (b) PIB2.5Cu, and (c) PIB5Cu within five successive loading-unloading tensile tests.

6. Thermal properties of PIB and PIBxCu (x=2.5, 5, 7.5, 10)

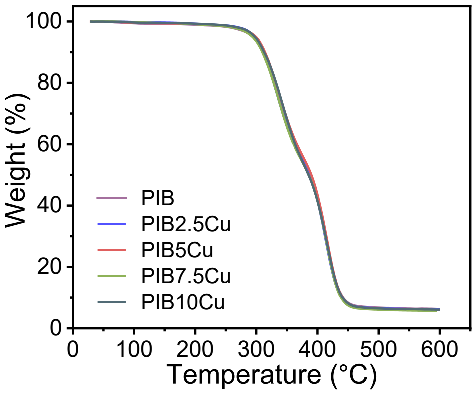

Figure S18 TGA curves of PIB and PIBxCu (x=2.5, 5, 7.5, 10) recorded under N2 flow (50 mL/min) with a heating rate of 20 °C/min.

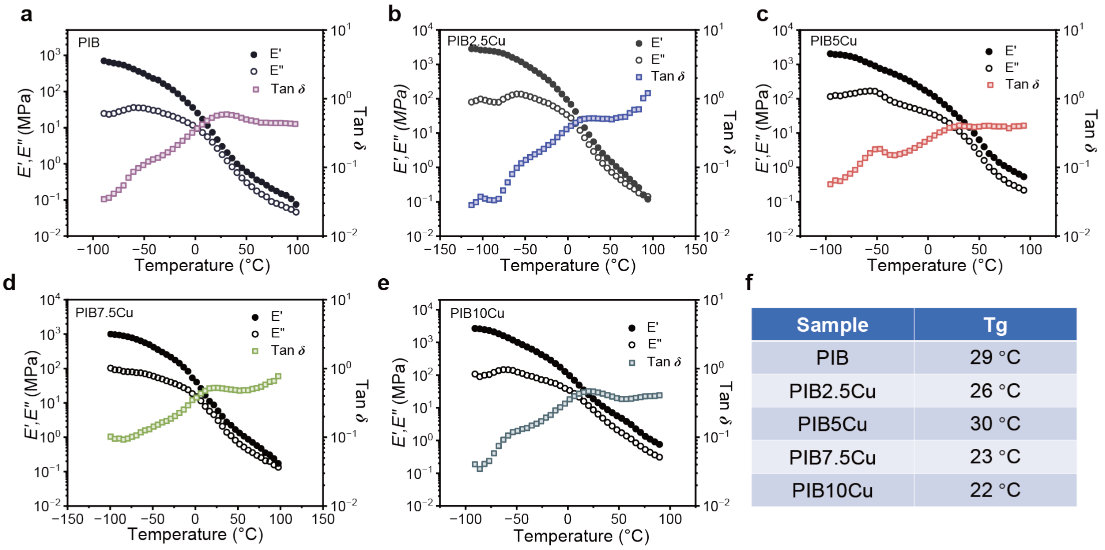

Sample	Tg
PIB	29 °C
PIB2.5Cu	26 °C
PIB5Cu	30 °C
PIB7.5Cu	23 °C
PIB10Cu	22 °C

Figure S19 Dynamic mechanical analysis (DMA) temperature sweeps of (a) PIB, (b) PIB2.5Cu, (c) PIB5Cu, (d) PIB7.5Cu, and (e) PIB10Cu from -100 to 100 °C. (f) Summary of Tg of all samples

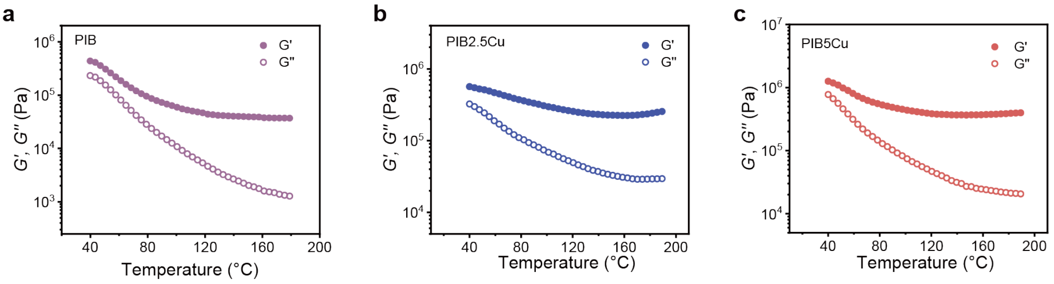

Figure S20 Temperature sweep rheological analysis of (a) PIB, (b) PIB2.5Cu, and (c) PIB5Cu in the range of 40-200 °C with a heating rate of 5.0 °C/min.

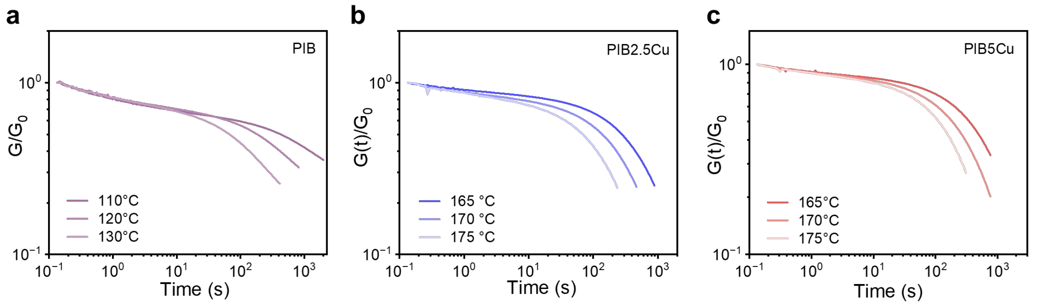

Figure S21 Stress relaxation curves of (a) PIB, (b) PIB2.5Cu, and (c) PIB5Cu.

7. Syntheses of small-molecule model

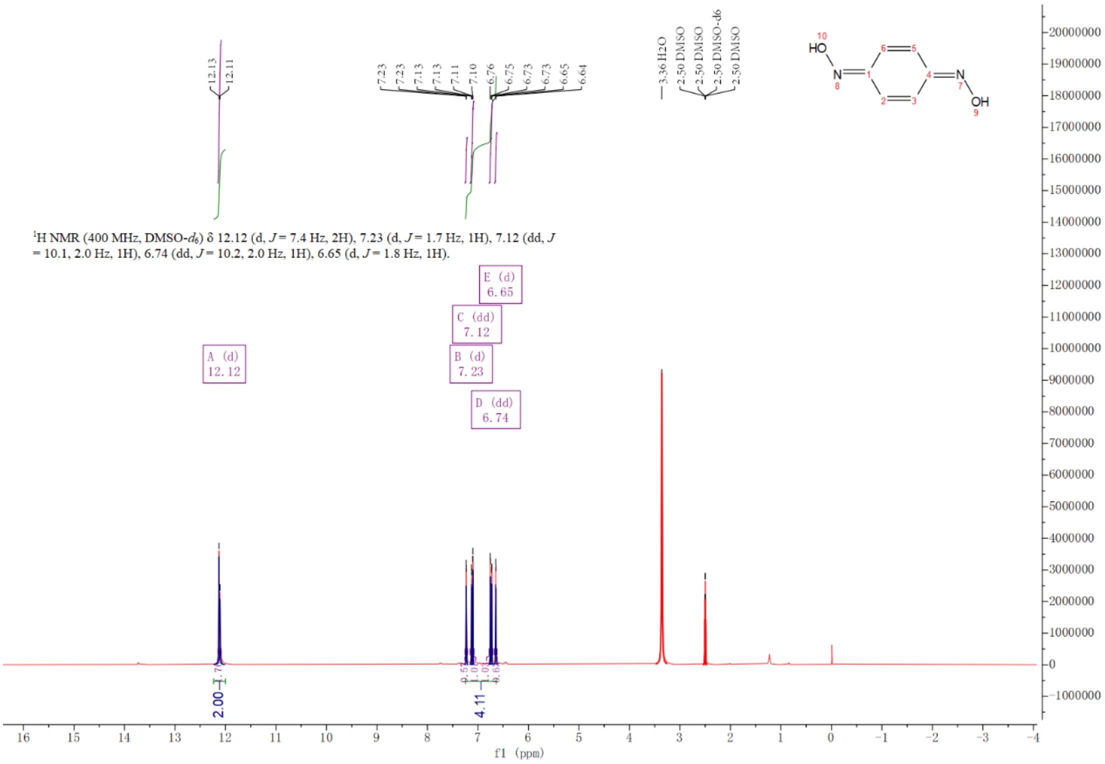

Figure S22 ¹H NMR spectrum of compound A (1,4-Benzoquinone pdioxime). ¹H NMR (600 MHz, DMSO-d_6) δ 12.12 (d, J = 7.4 Hz, 2H), 7.23 (d, J = 1.7 Hz, 1H), 7.12 (dd, J = 10.1, 2.0 Hz, 1H), 6.74 (dd, J = 10.2, 2.0 Hz, 1H), 6.65 (d, J = 1.8 Hz, 1H).

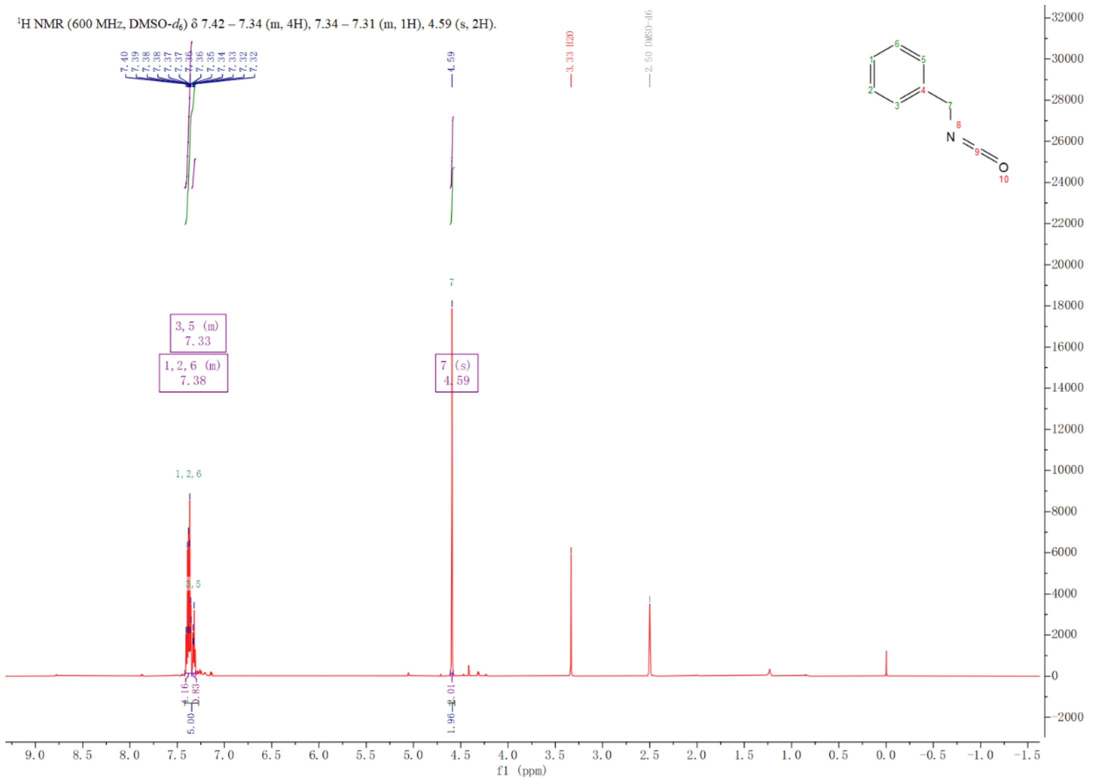

Figure S23 ^{1}H NMR spectrum of compound B (benzyl isocyanate). ^{1}H NMR (600 MHz, DMSO-d_6) δ 7.42 – 7.34 (m, 4H), 7.34 – 7.31 (m, 1H), 4.59 (s, 2H).

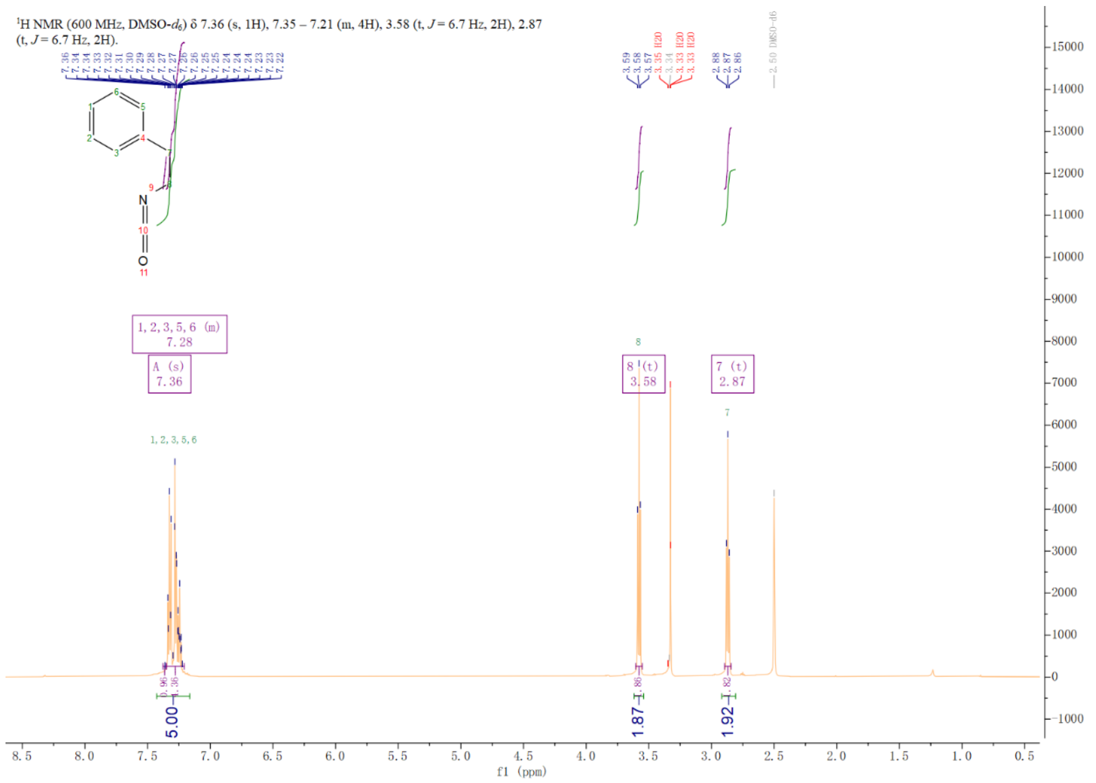

Figure S24 ^{1}H NMR spectrum of compound C (phenethyl isocyanate). ^{1}H NMR (600 MHz, DMSO-d_6) δ 7.36 (s, 1H), 7.35 – 7.21 (m, 4H), 3.58 (t, J = 6.7 Hz, 2H), 2.87 (t, J = 6.7 Hz, 2H).

Synthesis of small-molecule model compound AB

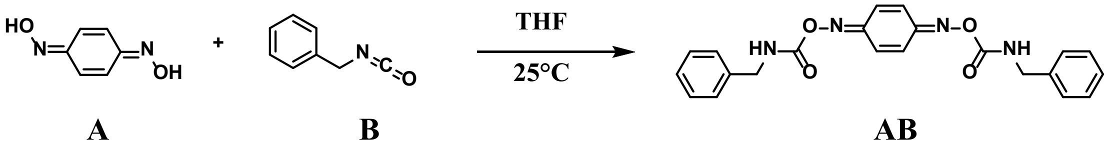

Figure S25 Synthesis of small-molecule model compound **AB**

1,4-Benzoquinone dioxime (compound A) was dissolved in THF (5 ml). Benzyl isocyanate (compound B) was added to react for 24 h at 25 °C under a nitrogen atmosphere. The resulting precipitate was added to n-hexane to generate a gray precipitate at -20 °C, and then it was dried at 30 °C under vacuum, finally compound ab was obtained.

Synthesis of small-molecule model compound AC

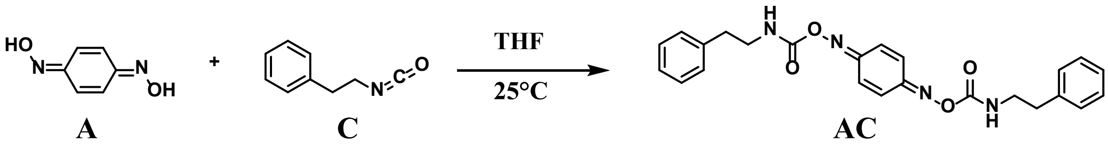

Figure S26 Synthesis of small-molecule model compound **AC**

1,4-Benzoquinone dioxime (compound A) was dissolved in THF (5 ml). Phenethyl isocyanate (compound C) was added to react at 25 °C under a nitrogen atmosphere. After 24 h, it was slowly added into n-hexane to generate a gray precipitate at -20 °C. Then the precipitate was dried at 30 °C under vacuum, at last compound ac was obtained.

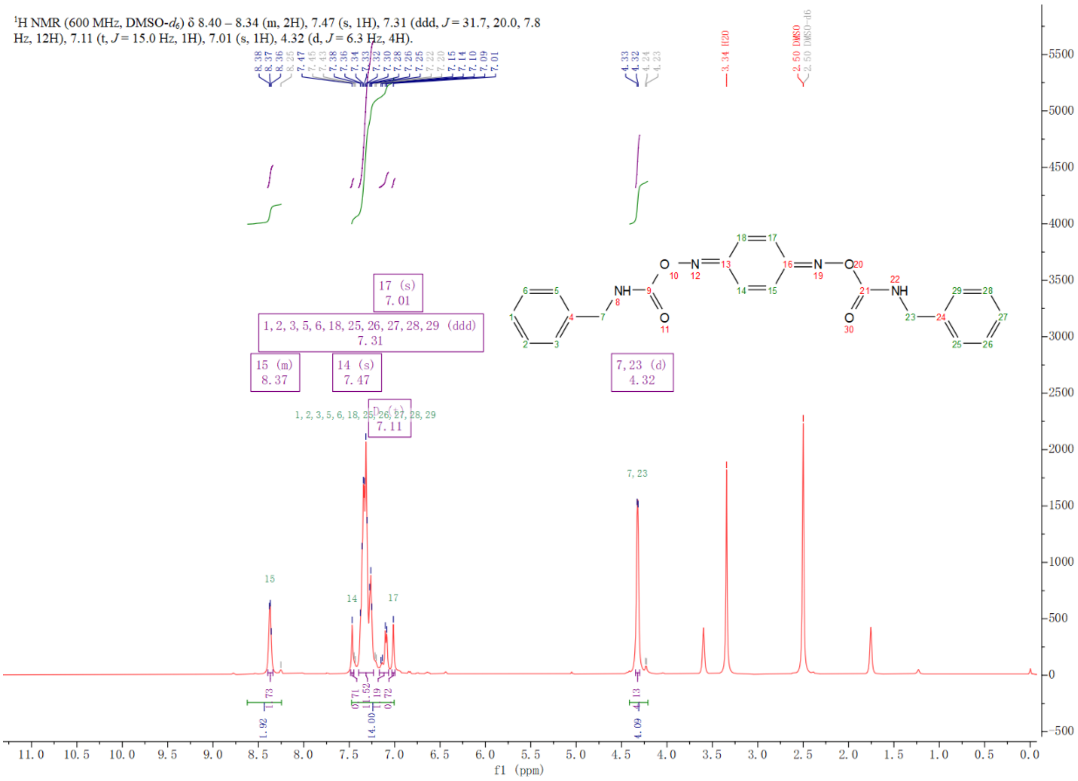

Figure S27 ^{1}H NMR spectrum of compound AB. ^{1}H NMR (600 MHz, DMSO-d_6) δ 8.40 – 8.34 (m, 2H), 7.47 (s, 1H), 7.31 (ddd, J = 31.7, 20.0, 7.8 Hz, 12H), 711 (t, J =

15.0 Hz, 1H), 7.01 (s, 1H), 4.32 (d, $J = 6.3$ Hz, 4H).

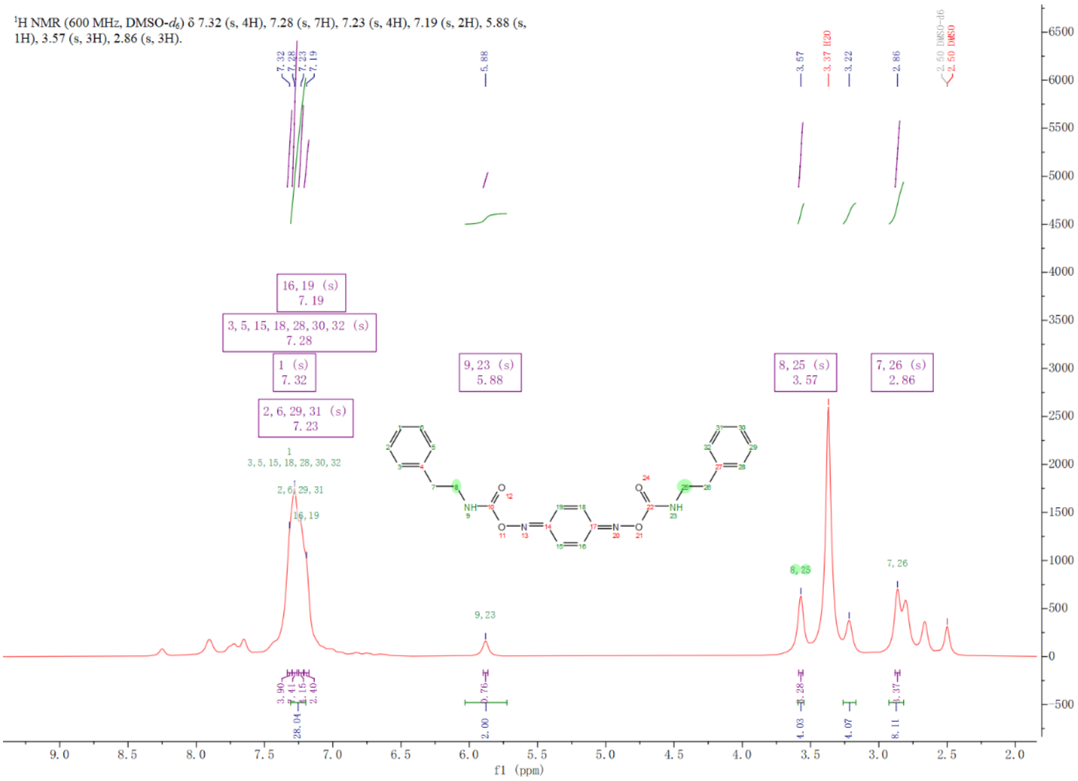

Figure S28 ^{1}H NMR spectrum of compound AC. ^{1}H NMR (600 MHz, DMSO-d_6) δ 7.32 (s, 4H), 7.28 (s, 7H), 7.23 (s, 4H), 7.19 (s, 2H), 5.88 (s, 1H), 3.57 (s, 3H), 2.86 (s, 3H).

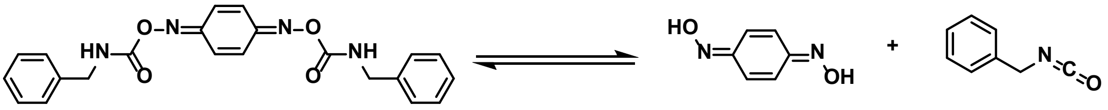

AB **A** **B**

Figure S29 *In situ* ^{1}H NMR spectra of compound **AB**.

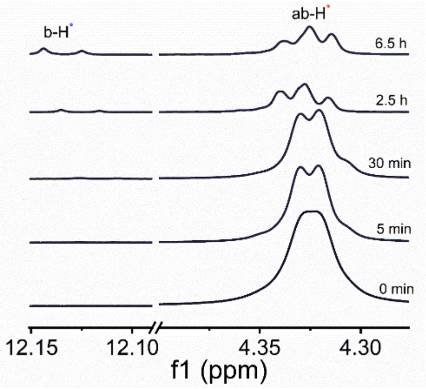

Figure S30 ^{1}H NMR spectra of compound AB at 100 °C, the proton peaks of B-H* appeared and their intensities gradually increased while the intensity of the proton peaks of AB-H* gradually decreased, indicating the generation of compound B and the

dissociation of compound AB.

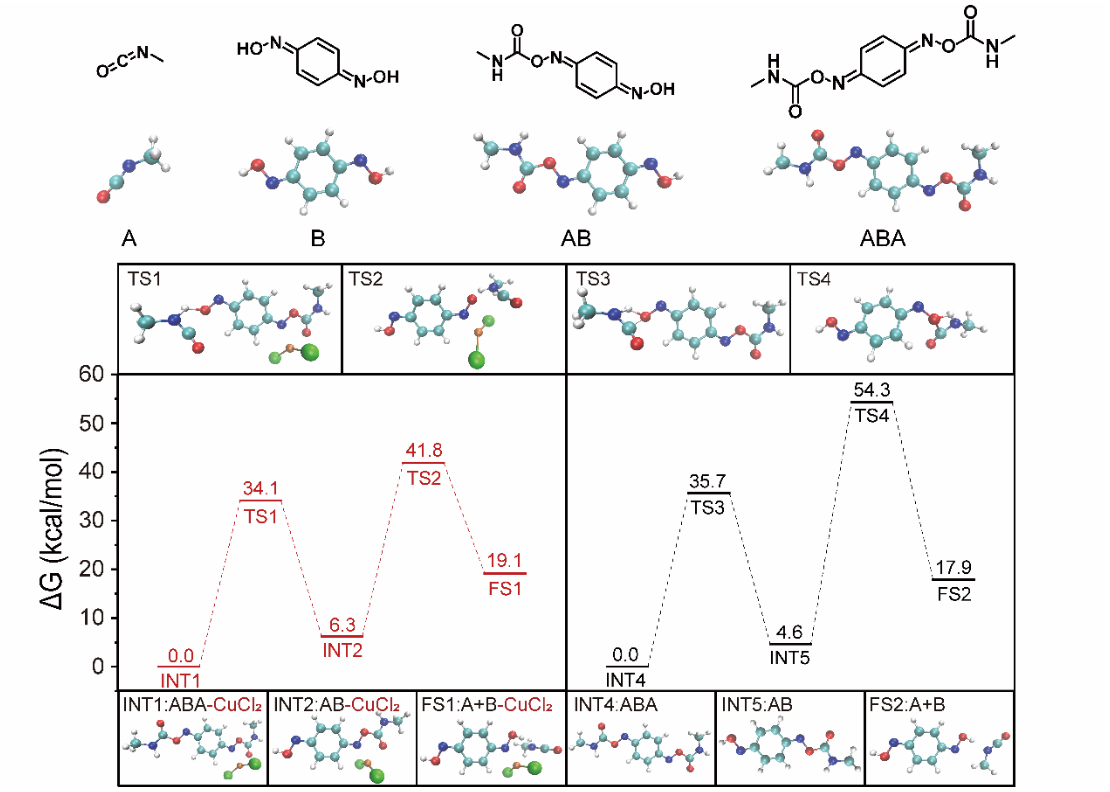

Figure S31 DFT-calculated energy surface for B (BQDO) conversion towards ABA (BQDU) with (left) and without (right) Cu(II) coordination. Introduction of copper ions reduced the activation energy required for the reaction. Geometries of the initial state (IS), transition state (TS), and final state (FS) of rate-determining steps (RDSs) in the first and second steps are shown above and below the free energy diagram, respectively. White, cyan, red, indigo, orange, and green balls represent H, C, O, N, Cu, and Cl atoms, respectively (color online).

8. Photothermal properties of PIB and PIBxCu (x=2.5, 5, 7.5, 10)

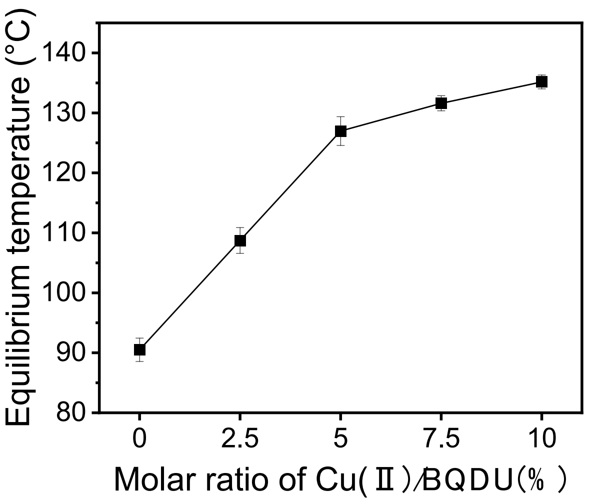

Figure S32 The relationship curve between photothermal equilibrium temperature and Cu(II) content.

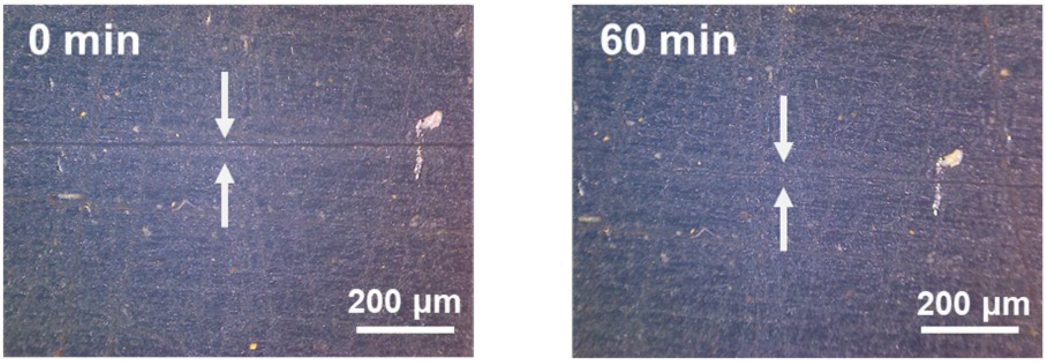

Figure S33 Optical microscopy images of PIB5Cu film after being scratched. It showed a rapid self-healing with an almost complete recovery within 1 h under NIR.

Reference

1. Stephens PJ, Devlin FJ, Chabalowski CF *et al.* Ab initio calculation of vibrational absorption and circular dichroism spectra using density functional force fields. *J Phys Chem* 1994; **98**: 11623-27.
2. Lee C, Yang W, Parr RG. Development of the colle-salvetti correlation-energy formula into a functional of the electron density. *Phys Rev B* 1988; **37**: 785-89.
3. Weigend F, Ahlrichs R. Balanced basis sets of split valence, triple zeta valence and quadruple zeta valence quality for h to rn: Design and assessment of accuracy. *Phys Chem Chem Phys* 2005; **7**: 3297-305.
4. Zheng J, Xu X, Truhlar DG. Minimally augmented karlsruhe basis sets. *Theor Chem Acc* 2010; **128**: 295-305.
5. Grimme S. Density functional theory with london dispersion corrections. *Wires*

Comput Mol Sci 2011; **1**: 211-28.
6. Frisch MJ, Trucks GW, Schlegel HB *et al.* Gaussian 16 rev. A.01. Wallingford, CT; 2016.
7. Perdew JP, Burke K, Ernzerhof M. Generalized gradient approximation made simple. *Phys Rev Lett* 1996; **77**: 3865-68.
8. Dolg M, Wedig U, Stoll H *et al.* Energy-adjusted ab initio pseudopotentials for the first row transition elements. *J Chem Phys* 1987; **86**: 866-72.
9. Fukui K. The path of chemical reactions - the irc approach. *Acc Chem Res* 1981; **14**: 363-68.
10. Weigend F, Ahlrichs R. Balanced basis sets of split valence, triple zeta valence and quadruple zeta valence quality for h to rn: Design and assessment of accuracy. *Phys Chem Chem Phys* 2005; **7**: 3297-305.
11. Bader RFW. A quantum theory of molecular structure and its applications. *Chem Rev* 1991; **91**: 893-928.
12. Cremer D, Kraka E. Chemical bonds without bonding electron density — does the difference electron-density analysis suffice for a description of the chemical bond? *Angew Chem Int Ed* 1984; **23**: 627-28.
13. Espinosa E, Lecomte C, Molins E. Experimental electron density overlapping in hydrogen bonds: Topology vs. Energetics. *Chem Phys Lett* 1999; **300**: 745-48.
14. Lu T, Liu Z, Chen Q. Comment on “18 and 12 – member carbon rings (cyclo[n]carbons) – a density functional study”. *Mat Sci Eng B* 2021; **273**: 115425.
15. Lu T, Chen Q. A simple method of identifying π orbitals for non-planar systems and a protocol of studying π electronic structure. *Theor Chem Acc* 2020; **139**.
16. Lu T, Chen Q. Independent gradient model based on hirshfeld partition: A new method for visual study of interactions in chemical systems. *J Comput Chem* 2022; **43**: 539-55.
17. Lu T, Chen F. Multiwfn: A multifunctional wavefunction analyzer. *J Comput Chem* 2012; **33**: 580-92.
18. Lu T. A comprehensive electron wavefunction analysis toolbox for chemists, multiwfn. *J Chem Phys* 2024; **161**: 082503.
19. Humphrey W, Dalke A, Schulten K. Vmd: Visual molecular dynamics. *J Mol Graph* 1996; **14**: 33-38.
20. Emamian S, Lu T, Kruse H *et al.* Exploring nature and predicting strength of hydrogen bonds: A correlation analysis between atoms-in-molecules descriptors, binding energies, and energy components of symmetry-adapted perturbation theory. *J Comput Chem* 2019; **40**: 2868-81.
