# Supplementary material for: Ultrastable, supertough and photohealable polymer
Source: Natl Sci Rev. 2025 Nov 27;13(1):nwaf521. doi: 10.1093/nsr/nwaf521 (PMC12796801; doi:10.1093/nsr/nwaf521)
Supplement: nwaf521_Supplemental_Files [file nwaf521_supplemental_files.zip › Teaser text.docx]

Leveraging coordination bonds to simultaneously polarize H-bonds, optimize π-π interactions and catalyze oxime-carbamate bonds achieves the simultaneous enhancement of elastomers’ mechanical properties, thermal stability, photothermal conversion efficiency and self-healing efficiency.
